# Identification of β‐galactosidases along the secretory pathway of *Nicotiana benthamiana* that collectively hamper engineering of galactose‐extended glycans on recombinant glycoproteins

**DOI:** 10.1111/pbi.70126

**Published:** 2025-05-07

**Authors:** Alex van der Kaaij, Myrna J. M. Bunte, Lisa Nijhof, Sanaz Mokhtari, Hein Overmars, Arjen Schots, Ruud H. P. Wilbers, Pieter Nibbering

**Affiliations:** ^1^ Laboratory of Nematology, Plant Sciences Department Wageningen University and Research Centre Wageningen The Netherlands

**Keywords:** *Nicotiana benthamiana*, β‐galactosidases, glycoengineering, Lewis X, β1,4‐galactosylation

## Abstract

Glycosylation is an important aspect for many biopharmaceuticals, including vaccines against parasitic helminths. Plants, especially *Nicotiana benthamiana*, have proven to be excellent production hosts for biopharmaceuticals with tailor‐made glycosylation. If desired, galactosylation can be introduced on biopharmaceuticals through co‐expression of the appropriate glycosyltransferase. However, achieving homogenous glycoforms with terminal galactose residues remains difficult as native *N. benthamiana* β‐galactosidases (NbBGALs) truncate these glycans. Recently, the first NbBGAL has been identified, but a knockout line was insufficient to achieve near complete galactosylation, suggesting that other enzymes could have similar activity. In this study, we selected 10 NbBGALs for further investigation into subcellular localization, *in vitro* and *in vivo* activity against β1,4‐linked galactose on N‐glycans and β1,3‐linked galactose on O‐glycans. We show that NbBGAL3B is localized in the apoplast and has similar specificity for β1,4‐linked galactose on N‐glycans as the previously identified NbBGAL1. In contrast, none of the selected NbBGALs cleaved β1,3‐linked galactose from O‐glycans besides BGAL1. In addition, we provide a novel strategy to achieve near complete galactosylation on galactosidase‐prone glycoproteins by using the protective capacity of the Lewis X motif and subsequent removal of the antennary fucose residues. Taken together, our results provide a broad view of the ability of NbBGALs to cleave galactoses and have identified NbBGAL3B as the second major contributor of undesired β‐galactosidase activity while engineering N‐glycans. This work lays the foundation for generating knockout lines that are devoid of undesired NbBGALs and therefore do not hamper the production of recombinant glycoproteins with galactose‐extended glycans.

## Introduction

*Nicotiana benthamiana* is known for its tolerance to engineering of the glycosylation pathway, in addition to high yields of recombinant proteins for therapeutic purposes (van der Kaaij *et al*., 2022). In the past, *N. benthamiana* has been utilized to produce large amounts of recombinant helminth glycoproteins with tailor‐made N‐glycans to study the immunomodulatory properties of these glycoproteins (Wilbers *et al*., 2017). Repurposing the *N. benthamiana* system to produce large quantities of recombinant helminth glycoproteins for vaccine purposes would greatly aid parasite vaccine development (Bunte *et al*., 2022).

Recently, *N. benthamiana* was used for the development of a recombinant vaccine against the cattle parasite *Ostertagia ostertagi*. Initially, a discrepancy was observed between native and recombinant glycoproteins during the vaccine development against *O. ostertagi*. Previous studies demonstrated that activation‐associated secreted protein 1 (Oo‐ASP‐1) purified from secreted material of adult parasites can provide protection (Geldhof *et al*., 2004; González‐Hernández *et al*., 2016; Meyvis *et al*., 2007). Initial tests with *P. pastoris* and insect cell recombinant Oo‐ASP‐1 proved unsuccessful (Geldhof *et al*., 2008; González‐Hernández *et al*., 2016). Therefore, recombinant Oo‐ASP‐1 was produced in *N. benthamiana* with its native N‐glycan composition including core α1,3‐ or α1,6‐fucosylation and β1,4‐linked galactosylation of a single antenna to restore the protective capacity of the vaccine. After mimicking the native N‐glycosylation composition of Oo‐ASP‐1, immunization of calves with *N. benthamiana*‐produced Oo‐ASP‐1 resulted in a significant reduction in faecal egg output, comparable to the protective efficacy of the native Oo‐ASP‐1 (Zwanenburg *et al*., 2023). *N. benthamiana*‐produced Oo‐ASP‐1 is therefore the first efficacious recombinant vaccine against the ruminant‐infecting helminth *Ostertagia ostertagi*, highlighting the importance of a versatile glycoprotein production platform.

While core fucosylation of Oo‐ASP‐1 N‐glycans can be achieved efficiently in *N. benthamiana*, the introduction of terminal galactose was limited (approximately 5%–10% of N‐glycans), which hampers efficient large‐scale production of Oo‐ASP‐1. Except for efficient galactosylation on antibodies, limited galactosylation is a common problem with *N. benthamiana*‐produced glycoproteins (e.g. α1‐antitrypsin) (Castilho *et al*., 2014; Kallolimath *et al*., 2018). Thus, improving galactosylation efficiency is of importance for the development of a wide range of plant‐produced biopharmaceuticals that rely on a specific glycan composition.

Incomplete galactosylation is likely caused by the presence of endogenous β‐galactosidases (BGALs) that cleave off galactose from secreted glycoproteins upon engineering. There are two groups of β‐galactosidases in plants, of which the largest group is the glycosylhydrolase family GH35 (Chandrasekar and Van Der Hoorn, 2016; Nibbering *et al*., 2020). The *Arabidopsis thaliana* genome contains 17 GH35 members, while the *N. benthamiana* genome contains more than 28 putative GH35 members, excluding allelic copies (Buscaill *et al*., 2019). The GH35 family members are exo‐β‐galactosidases involved in the modification of terminal galactose from cell wall and glycan moieties in the apoplastic space (Chandrasekar and Van Der Hoorn, 2016). These modifications are involved in developmental processes, like fruit ripening (Smith and Gross, 2000), flowering (Sampedro *et al*., 2012) and seed coat development (Dean *et al*., 2007). Furthermore, BGALs play an important role in plant defence against bacterial pathogens (Buscaill *et al*., 2019). A small number of GH35 family members have been functionally characterized, which has mainly been performed in the model organism *A. thaliana*. The majority of characterized GH35 enzyme members exhibit activity against terminal β1,3‐ and β1,4‐galactose linkages (AtBGAL1‐5), while AtBGAL12 exhibits activity against terminal β1,3‐, β1,4‐ and β1,6‐galactose linkages (Gantulga *et al*., 2008, 2009). Interestingly, an AtBGAL8 orthologue from radish (RsBGAL1) does not hydrolyse β1,4‐galactose linkages and preferentially removes terminal β1,3‐ and β1,6‐galactose linkages (Kotake *et al*., 2005). However, it remains unknown whether orthologues of these BGALs in *N. benthamiana* (NbBGALs) are involved in the removal of terminal galactose from glycans of plant‐produced biopharmaceuticals.

Previous research by Kriechbaum *et al*. (2020) identified NbBGAL1 (AtBGAL8 orthologue) as a driver of undesired β‐galactosidase activity in the apoplast using RNAi‐mediated silencing, CRISPR‐mediated gene editing and *in vitro* activity assays with apoplast fluid. NbBGAL1 was able to cleave β1,4‐galactose from N‐glycans as well as β1,3‐galactose from mucin type O‐glycans in *in vitro* activity assays. RNAi and CRISPR editing of NbBGAL1 resulted in increased levels of galactosylated N‐glycans on several target proteins, although not completely. This suggests that additional NbBGALs likely contribute to the removal of terminal galactoses on glycans. Therefore, it remains unknown which specific BGALs in *N. benthamiana* (NbBGALs) are collectively involved in the processing of glycans on plant‐produced biopharmaceuticals.

This study aimed to provide a broad screening of NbBGALs to identify enzymes that trim off terminal galactoses to advance glycoengineering efforts in *N. benthamiana*. We selected numerous β‐galactosidase sequences from *N. benthamiana* based on a bioinformatic analysis and subsequently cloned the NbBGAL open reading frames. The NbBGAL genes were first investigated for their subcellular localization to confirm localization along the secretory pathway. Afterwards, all NbBGALs were screened for *in vivo* activity against β1,4‐linked galactose on N‐glycans and β1,3‐linked galactose on O‐glycans. Finally, we show that near full mono‐ or di‐galactosylation can be achieved through the generation of the Lewis X (Galβ1‐4(Fucα1‐3)GlcNAc) motif and the subsequent removal of the antennary fucose residue, leaving a terminal β1,4‐linked galactose. Our findings encompass a functional characterization of numerous NbBGALs against galactosylated glycoproteins and the identification of NbBGAL1 (renamed NbBGAL8) and NbBGAL3B responsible for the undesired galactosidase activity along the secretory pathway. These results enable a targeted genome editing approach to further enhance the capabilities of *N. benthamiana* as a production platform for glycoproteins with a tailored glycan composition.

## Results

### Bioinformatic analysis for NbBGAL selection

**Figure 1:**
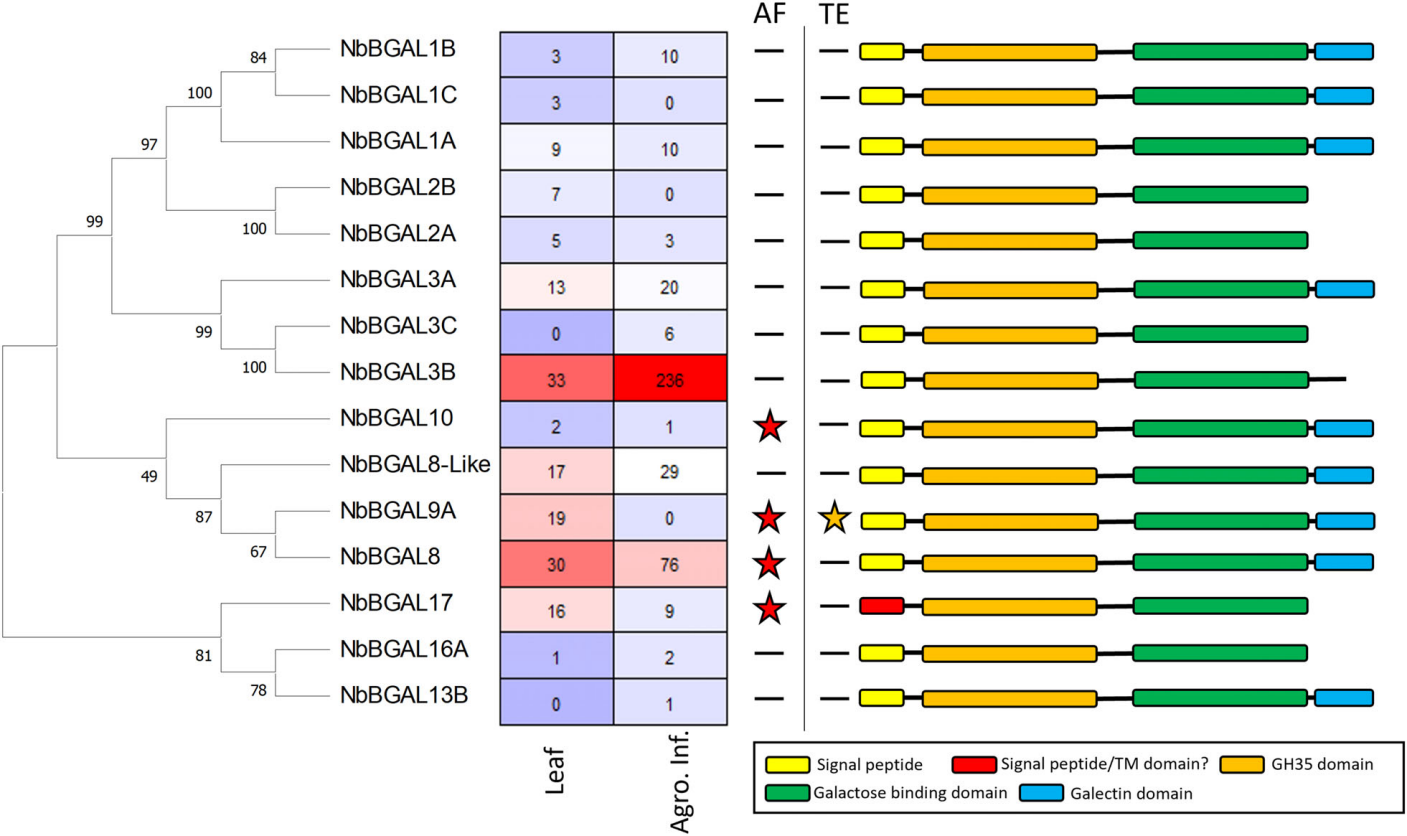
Bioinformatic analysis of highly expressed NbBGALs in leaves. Different NbBGALS were identified in genome databases and aligned by ClustalW. Phylogenetic trees were constructed using the maximum likelihood method of MEGA‐X in default mode, with a bootstrap test of 1000 replicates. The numbers beside the branches correspond to % bootstrap values. For each NbBGAL, the expression levels (transcripts per million) in leaves (Benth genome) or in leaves after agroinfiltration (Grosse‐Holz *et al*., 2018) are indicated in a heatmap. The presence of NbBGALs in apoplast fluid (AF) and/or total extract (TE) in the proteomic analysis of Grosse‐Holz *et al*. (2018) is indicated with stars. The different protein domains of each NbBGAL based on InterPro prediction are illustrated.

*Nicotiana benthamiana* is a tetraploid, making it complex to construct a complete genome and to fully identify gene families. For this reason, we used the BLAST tools from Sol Genomics Network (SGN) (Fernandez‐Pozo *et al*., 2015) and Benthgenome (Ranawaka *et al*., 2023) to search for putative NbBGALs using the 17 available amino acid sequences of *A. thaliana* BGALs. The NbBGALs identified in both databases were compared for expression levels in uninfiltrated leaf tissue and upon *Agrobacterium*‐mediated transformation of the leaf tissue. NbBGALs showing expression levels under both conditions were selected and named based on their closest *Arabidopsis* orthologue. A phylogenetic tree was constructed in combination with an expression heatmap displaying the differential expression in leaf tissue (Figure 1). In addition, we displayed whether the selected NbBGALs were previously identified in the proteome of leaf tissue and apoplastic fluid upon agroinfiltration (Grosse‐Holz *et al*., 2018), and included the predicted protein domains as inferred using InterPro (Figure 1). These data show that NbBGAL3A, NbBGAL3B, NbBGAL8 (previously reported as BGAL1), NbBGAL8‐like, NbBGAL9A and NbBGAL17 have the highest expression in leaves (Figure 1). In addition, NbBGAL8, NbBGAL9A, NbBGAL10 and NbBGAL17 have previously been detected in apoplastic fluid after Agrobacterium‐mediated transformation. All identified NbBGALs have a predicted signal peptide (Figure 1), which suggests that these enzymes go through the secretory pathway and possibly end up in the apoplastic space, where they are expected to process galactosylated glycans. We decided to continue with a selection of the NbBGALs based on their homology, expression levels in leaf tissue and previously identified apoplastic localization. In addition, we included several NbBGALs based on the highest expression in the remaining clades of the phylogenetic tree. The selected NbBGALs included the isoforms: 1B, 2A, 3A, 3B, 8, 9A, 8‐like, 10, 16A and 17.

### Subcellular localization of NbBGALs

**Figure 2:**
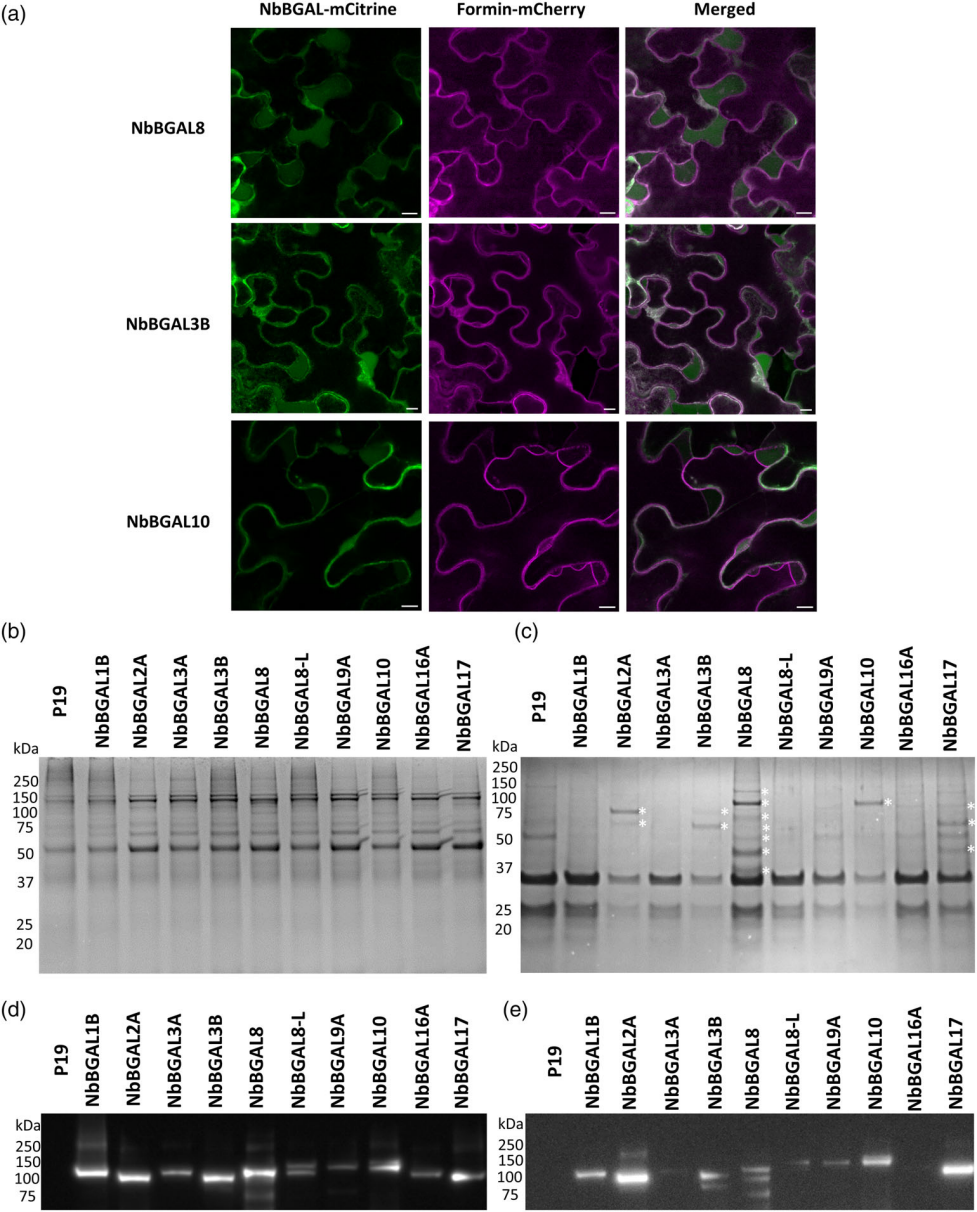
Localization of mCitrine‐tagged NbBGALs. (a) Confocal laser scanning microscope images of leaf epidermal cells transiently expressing different NbBGAL‐mCitrine fusions and the plasma membrane marker formin‐mCherry. Leaves were treated with 500 mM NaCl to induce plasmolysis and to allow plasma membranes to collapse inside the cell. The mCitrine and mCherry signals were captured using a Stellaris 5 Confocal LSM (Leica). Scalebar is 10 μm. (b–e) NbBGAL‐mCitrine fusions were transiently expressed and crude extracts (b and d) or apoplastic fluids (c and e) content were extracted and visualized by Coomassie blue staining (b and c) and western blotting (d and e) with an anti‐GFP antibody. White asterisks (*) indicate fragments not found in the P19 control in the Coomassie blue staining.

To investigate the subcellular localization of the selected NbBGALs, we first amplified cDNAs from leaf tissue and fused this DNA with a C‐terminal mCitrine (yellow fluorescent protein) tag. The NbBGAL‐mCitrine constructs were transiently expressed together with a mCherry‐tagged formin cell membrane marker (Favery *et al*., 2004). Under normal conditions, it is difficult to distinguish apoplastic and membrane localization with confocal microscopy, which is why we induced plasmolysis with a high salt treatment. Under these conditions, the plant cell loses water, causing the plasma membrane to retract inward and making the apoplastic localization more pronounced. Under these conditions, all BGALs, except BGAL16A and BGAL17, revealed an apoplastic signal (Figure 2a, Figure S1). To confirm apoplastic localization, we overexpressed the mCitrine‐fused NbBGALs, isolated apoplastic and intracellular proteins, and analysed them with Coomassie staining and anti‐GFP western blot (Figure 2c–e). Western blot data revealed that all selected NbBGALs exhibit a signal in both the crude and apoplastic extracts, although NbBGAL3A and NbBGAL16A only have a weak signal in the apoplastic extracts. Coomassie stained gels showed that truncated versions of NbBGAL2A, NbBGAL3B, NbBGAL10 and NbBGAL17 are present in the apoplastic extracts. Most of the truncated versions lacked the mCitrine tag since these were not visible on the western blots. Interestingly, NbBGAL17 was detected as several truncated forms in the apoplastic extracts and exhibited a strong signal on the western blot but displays a fragmented intracellular confocal signal suggestive of ER and Golgi localization. These data indicated that NbBGAL17 was located in the apoplastic space but that the apoplastic signal was masked by the intracellular signal. In summary, all the identified NbBGALs, except NbBGAL16A, showed an apoplastic localization, making them possible N‐glycan modifying enzymes.

### Overexpression of NbBGAL3B and NbBGAL8 results in cleavage of β1,4‐linked galactose from engineered N‐glycans

We hypothesized that besides the previously characterized NbBGAL8 (Kriechbaum *et al*., 2020), additional NbBGALs are involved in cleaving β1,4‐linked galactose from N‐glycans. To investigate this, we screened the NbBGALs for activity against β1,4‐linked galactose on N‐glycans. We co‐expressed SmKappa‐5 as a carrier protein in ΔXT/FT *N. benthamiana* plants together with SialDrGalT (under the control of the 35S promoter) to engineer galactosylated N‐glycans, resulting in hybrid‐type N‐glycans containing a single galactose‐extended antenna. SmKappa‐5 was purified from the apoplast via Ni‐NTA chromatography and analysed for glycan composition.

**Figure 3:**
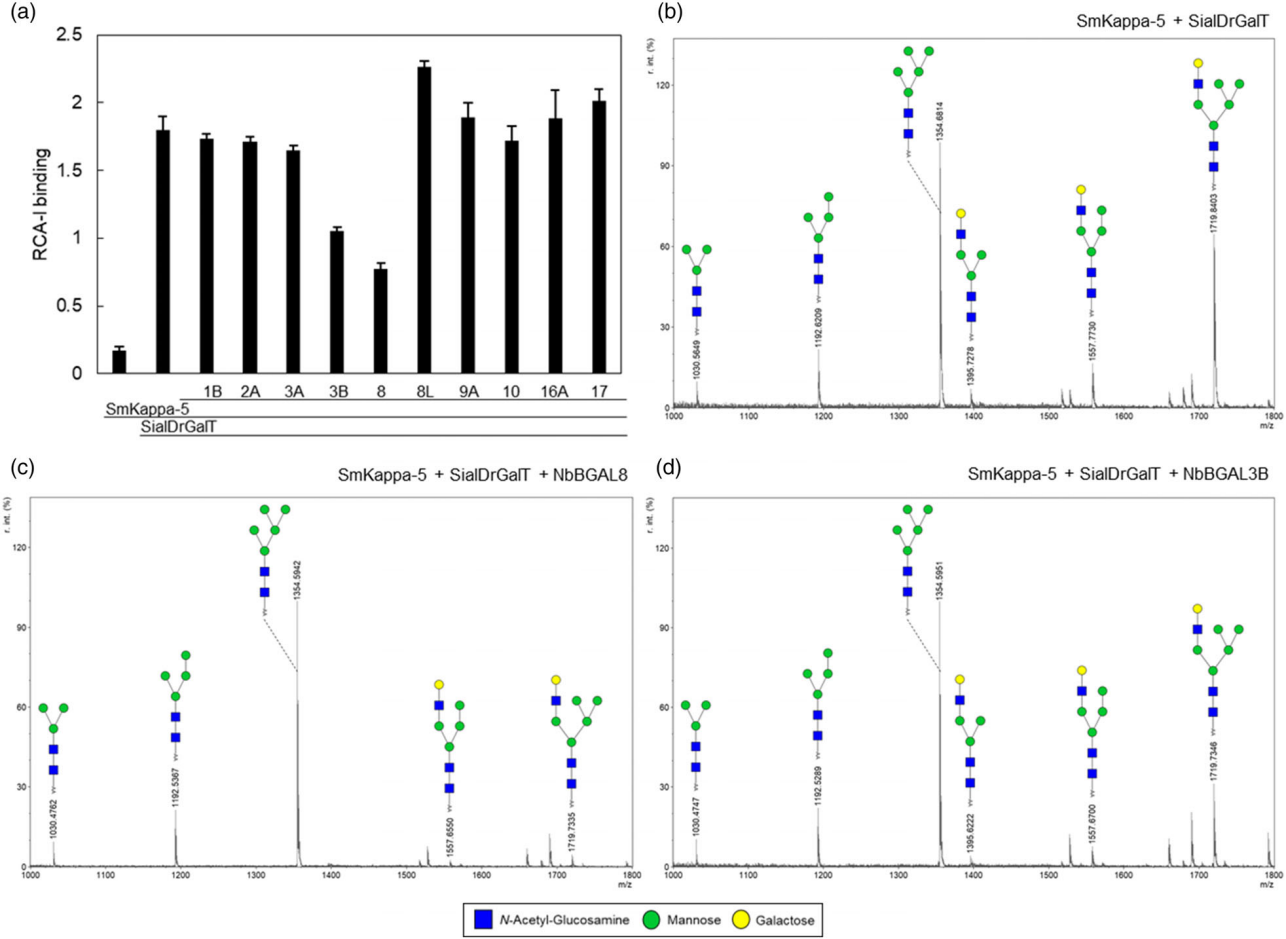
*In vivo c*leavage of terminal β1,4‐linked galactose from N‐glycans by NbBGALs. SmKappa‐5 was co‐expressed with 35S:SialDrGalT and different NbBGALs in ΔXT/FT *N. benthamiana* plants. (a) A preliminary screening was conducted on purified SmKappa‐5 using a Ricinus Communis Agglutinin I (RCA‐I) binding assay for detection of terminal galactose on the N‐glycans. Lectin binding was measured as absorbance at 450 nm. (b–d) MALDI‐TOF‐MS N‐glycan analysis of purified SmKappa‐5 co‐expressed with 35S:SialDrGalT only (b) or combined with co‐expression of NbBGAL8 (c) or NbBGAL3B (d). All samples were treated with β‐*N*‐acetyl‐glucosaminidase to confirm the presence of galactose‐extended antennae. Peaks of interest were labelled with the corresponding N‐glycan structures.

The level of β1,4‐linked galactose on SmKappa‐5 N‐glycans was first assessed using an RCA‐I lectin‐binding assay (Figure 3a). Purified SmKappa‐5 without engineering only showed a low background signal, while co‐expressing SialDrGalT to engineer galactose‐extended N‐glycans allowed strong RCA‐I binding. A reduction of RCA‐I binding was then observed when NbBGAL3B and NbBGAL8 were co‐expressed, indicating that these enzymes could cleave off the engineered β1,4‐linked galactose from N‐glycans. For the other NbBGALs, we did not observe any reduction in RCA‐I binding. To confirm the ability of NbBGAL3B to cleave β1,4‐linked galactose, MALDI‐TOF‐MS analysis was performed on PNGase F released N‐glycans from purified SmKappa‐5 (Figure 3b–d, Figure S2). The released N‐glycans were treated with a β‐*N‐*acetyl‐glucosaminidase to confirm the presence of terminal galactose in the antenna. Co‐expression of SmKappa‐5 with SialDrGalT revealed the baseline galactosylation level, with degalactosylated (m/z 1354) and galactosylated (m/z 1719) Man5 hybrid N‐glycan being dominant in the MS spectra (Figure 3b). Minor peaks included paucimannosidic (m/z 1030), Man4 (m/z 1192) and single antenna versions of these N‐glycans with terminal galactose (m/z 1395 and 1557). Furthermore, we found many unidentified peaks with larger m/z values than expected, which were confirmed to be N‐glycans with additional non‐engineered arabinofuranose and galactose residues (Figure S3). MS analysis also confirmed that upon co‐expression of NbBGAL8 the galactosylated N‐glycan peaks were almost completely gone (Figure 3c), indicating the potency of NbBGAL8 to cleave β1,4‐linked galactose when overexpressed in leaves. Co‐expression of NbBGAL3B (Figure 3d) also resulted in a strong reduction of galactosylated hybrid N‐glycans. All three galactose‐containing peaks were reduced, albeit not to the same extent when compared to NbBGAL8. The other NbBGALs were also analysed using MALDI‐TOF‐MS but did not reveal degalactosylation upon overexpression (Figure S2). Altogether, we conclude that besides NbBGAL8, NbBGAL3B also drives degalactosylation of engineered N‐glycans *in planta*.

### *In vitro* activity of NbBGALs

**Figure 4:**
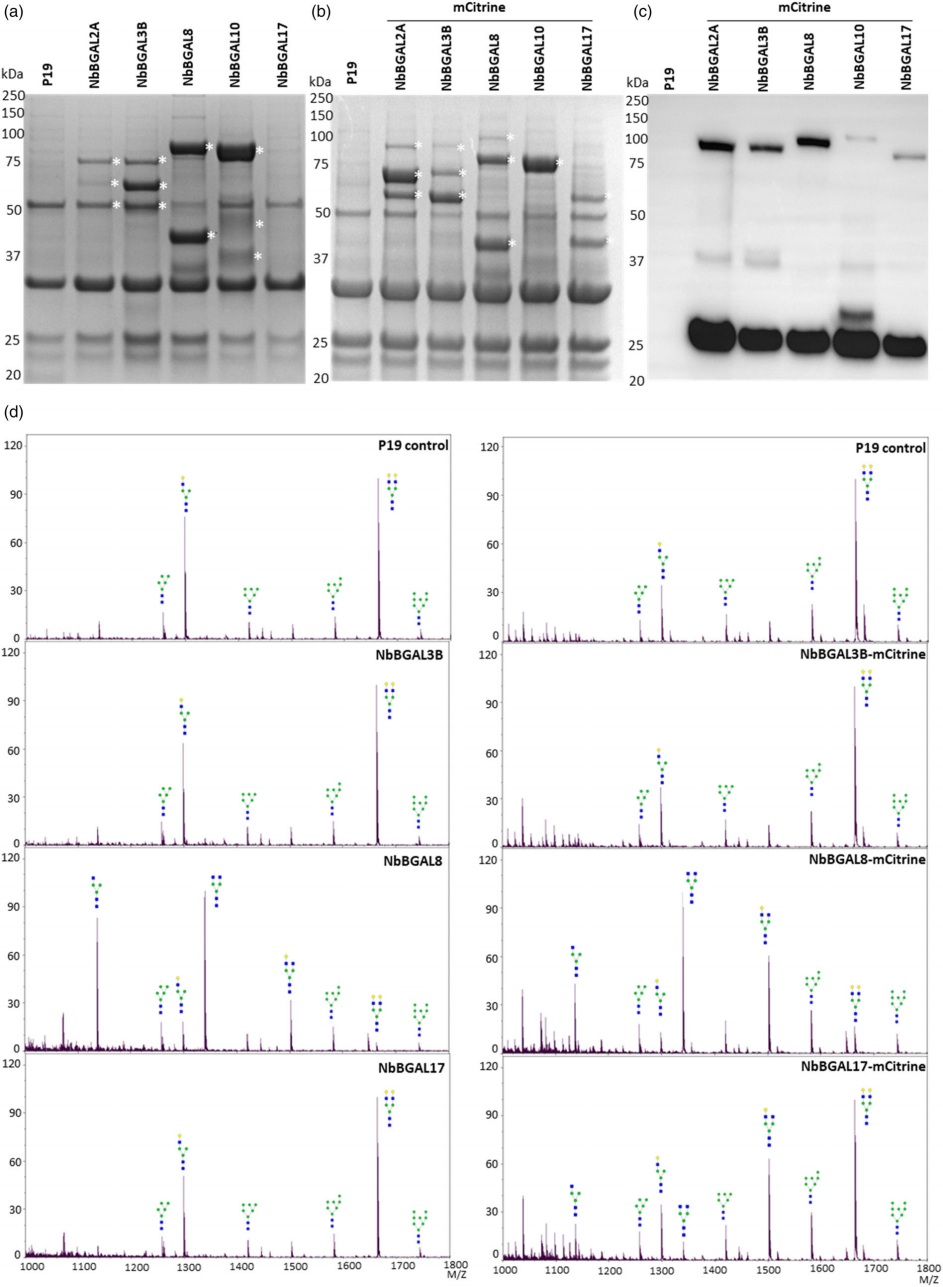
*In vitro* β‐galactosidase activity of NbBGALs. (a–c) NbBGALs with or without mCitrine fusion were expressed and isolated from the apoplastic space. The extracts were analysed by Coomassie blue staining (a and b) or by western blotting using an anti‐GFP antibody (c). White asterisks (*) indicate fragments not found in the P19 control in the Coomassie blue staining. (d) MALDI‐TOF‐MS N‐glycan analysis of *in vitro* glyco‐engineered SmKappa‐5 after treatment with apoplast fluids from p19 control infiltrations or apoplast fluids upon overexpression of NbBGAL3B, NbBGAL8 or NbBGAL17 (with or without mCitrine fusion).

To confirm the results obtained *in vivo*, we performed *in vitro* galactosidase assays to test for differences in specificity of the identified NbBGALs. Both the untagged and mCitrine‐tagged versions of NbBGAL2A, NbBGAL3B, NbBGAL8, NbBGAL10 and NbBGAL17 were overexpressed in wild‐type *N. benthamiana* plants, and the apoplastic fluid was isolated from those plants (Figure 4a–c). The Coomassie results show that all the untagged versions, except NbBGAL17, are extractable from the apoplastic space, while the mCitrine‐tagged versions are all extractable. An anti‐GFP western blot indicates that full‐length enzymes entered the apoplastic space, but that most of the extractable enzymes had lost their mCitrine tag.

The apoplastic fluids were used to test the enzymatic activity of the selected NbBGALs. First, the enzymes were tested for their activity on galactobiose substrates with β1,3‐, β1,4‐ or β1,6‐linkages to show differences in specificity (Figure S4). The results indicated that NbBGAL2A preferentially digests β1,3‐ and β1,4‐galactobiose linkages, with lower activity on β1,6‐galactobiose. NbBGAL8 was shown to efficiently digest β1,3‐ and β1,6‐galactobiose, with a lower activity on β1,4‐galactobiose. In addition, NbBGAL10 was able to digest β1,3‐galactobiose and showed no visible activity on the other substrates, while NbBGAL3B and NbBGAL17 had no visible activity on all three galactobiose substrates.

**Figure 5:**
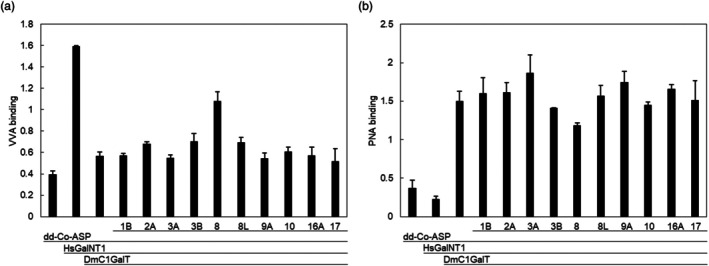
Mucin type O‐glycan synthesis is marginally affected by NbBGALs *in vivo*. dd‐Co‐ASP was co‐expressed with HsGalNT1, DmC1GalT and different NbBGALs in wild‐type *N. benthamiana* plants. (a) A lectin‐based binding assay was conducted on apoplast fluid containing dd‐Co‐ASP using Vicia Villosa Lectin (VVA) for the detection of a single α‐linked *N*‐acetylgalactosamine (Tn antigen). (b) A lectin‐binding assay with Peanut Agglutinin (PNA) was conducted for the detection of galactose(β1,3)*N*‐acetylgalactosamine (T‐antigen). Lectin binding was measured as absorbance at 450 nm.

To further test their specificity on terminal β1,4‐galactosylated N‐glycans, we synthesized a non‐hybrid N‐glycan substrate to test the BGAL enzymes. We expressed and purified SmKappa‐5 from ΔXT/FT *N. benthamiana* plants, generating GnM3 and GnGn N‐glycan structures (Figure S5a). The purified SmKappa‐5 was then modified with an *E. coli‐*produced β1,4‐galactosyltransferase to generate a fully galactosylated version of SmKappa‐5 with single and double antennas (Figure S5b,c). The galactosylated N‐glycan substrate was then used to test for galactosidase activity in apoplastic extracts containing the different NbBGALs with and without the mCitrine tag. NbBGAL8 showed activity on single and double galactose‐extended N‐glycans, as previously reported (Kriechbaum *et al*., 2020). Only the mCitrine‐tagged version of NbBGAL17 was capable of modifying galactosylated N‐glycans *in vitr*o, whereas the apoplast fluid with untagged NbBGAL17 showed no activity, likely due to the absence of the enzyme in the apoplast fluid (Figure 5d, Figure S6). NbBGAL10 (without tag) showed mild *in vitro* activity towards N‐glycans, whereas NbBGAL2A exhibited no activity on β1,4‐galactosylated N‐glycans. This contrasts with the high activity towards β1,4‐galactobiose (Figure S4), highlighting the different enzymatic specificities of this enzyme. Surprisingly, NbBGAL3B did not show *in vitro* activity towards different galactobiose substrates, nor towards galactosylated N‐glycan substrates.

To verify if the mCitrine tag could affect enzyme activity, we repeated the *in vivo* experiment with SmKappa‐5, 35S:SialDrGalT and transient overexpression of mCitrine‐fused NbBGALs. We used MALDI‐TOF‐MS to evaluate the galactosylation levels of SmKappa‐5 (Figure S7). MS data revealed that NbBGAL3B, NbBGAL8 and NbBGAL17 were able to modify the terminal β1,4‐galactose on N‐glycans of SmKappa‐5 *in planta*, unlike NbBGAL2A and NbBGAL10, which showed no reduced levels of galactosylation in comparison to the control. This suggests that the mCitrine fusion does not affect the activity of the enzymes, while merely affecting the secretion of BGAL17 into the apoplast.

### β1,3‐linked galactose of engineered O‐linked glycans is largely unaffected by NbBGALs

Next, we investigated whether any of the tested NbBGALs could cleave β1,3‐linked galactose that is commonly found on O‐glycans. Previous research reported that NbBGAL8 could cleave the aforementioned O‐glycans (Kriechbaum *et al*., 2020). Therefore, we applied a similar approach as with the N‐glycans, engineering the core 1 O‐glycan (containing the β1,3‐linked galactose) and co‐expressing the NbBGALs. As a carrier protein for O‐glycans, we used the double‐domain activation‐associated secreted protein from *Cooperia oncophora* (dd‐Co‐ASP), which contains a linker region rich in threonines and prolines (Figure S8). This linker region was O‐glycosylated by co‐expressing *Homo sapiens* polypeptide:*N*‐acetylgalactosaminyltransferase 1 (HsGalNT‐1) and *Drosophila melanogaster* core 1 β1,3‐galactosyltransferase (DmC1GalT), resulting in core 1 O‐glycan synthesis. After production in WT *N. benthamiana* plants, the apoplast fluids of each sample were screened with two lectin‐binding assays, using *Vicia Villosa* Agglutinin (VVA) and Peanut Agglutinin (PNA) lectin‐binding α‐linked GalNac and core 1 O‐glycan (Gal‐GalNAc), respectively.

While screening with the VVA lectin, only a background signal was observed when expressing dd‐Co‐ASP (Figure 5a). Upon engineering O‐glycans by co‐expressing HsGalNT‐1, strong binding was observed for the VVA lectin. Co‐expression of DmC1GalT to engineer the core 1 O‐glycan caused the VVA binding to drop back to background levels, indicating that β1,3‐galactose extension prevents VVA from binding to the O‐glycans. Then, we co‐expressed various NbBGALs, and an increase in VVA lectin binding was only observed upon co‐expression of NbBGAL8. This indicates that the terminal β1,3‐galactose is only removed to some extent by NbBGAL8. Next, we continued with a PNA lectin‐binding assay (Figure 5b), which binds to the core 1 O‐glycan. A background signal was observed when only expressing dd‐Co‐ASP. Co‐expressing HsGalNT‐1 to engineer GalNAc did not result in stronger PNA binding, whereas the co‐expression of both HsGalNT1 and DmC1GalT strongly increased PNA binding. Subsequently, we co‐expressed various NbBGALs, and a decrease in PNA lectin binding was only observed upon co‐expression of NbBGAL8, Thus confirming that the terminal β1,3‐galactose is removed only to some extent by NbBGAL8.

### Production of glycoproteins with near complete galactosylation via synthesis of Lewis X

**Figure 6:**
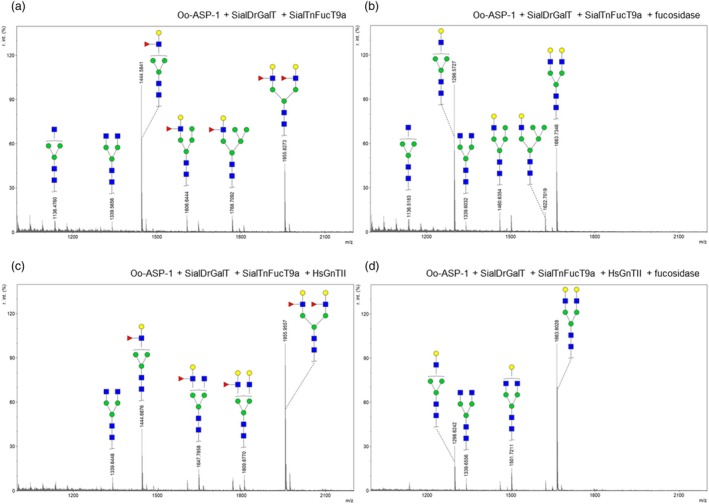
Efficient galactosylation through Lewis X engineering in *N. benthamiana* and *in vitro* removal of antennary fucose. Oo‐ASP‐1 was co‐expressed with GPAII:sialDrGalT and SialTnFucT9a in ΔXT/FT *N. benthamiana* plants to enable Lewis X synthesis on N‐glycans of Oo‐ASP‐1. (a, b) MALDI‐TOF MS N‐glycan profiles of purified Oo‐ASP‐1 upon co‐expression of GPAII:SialDrGalT and SialTnFucT9a before (a) and after (b) *in vitro* fucosidase treatment. (c, d) MALDI‐TOF MS N‐glycan profiles of Oo‐ASP‐1 upon co‐expression of GPAII:SialDrGalT, SialTnFucT9a and HsGnTII (for synthess of diantennary N‐glycans) before (c) and after (d) *in vitro* fucosidase treatment.

The galactosylation of glycoproteins with surface‐exposed N‐glycans in *N. benthamiana*, such as SmKappa‐5 and the vaccine candidate Oo‐ASP‐1, is very limited (Zwanenburg *et al*., 2023). To produce galactosylated glycoproteins efficiently, we devised a Lewis X engineering strategy. Glycoengineering of the galactose‐bearing glycan structure Lewis X (Galβ1‐4(Fucα1‐3) GlcNAc) can be efficiently achieved using SialDrGalT in combination with hybrid α1,3‐fucosyltransferase IXa from *Tetraodon nigriviridus* (SialTnFUT9a) (Wilbers *et al*., 2017). This suggests that the presence of the antennary fucose of Lewis X shields the N‐glycans against endogenous NbBGALs. Therefore, we engineered Lewis X on the N‐glycans of Oo‐ASP‐1 through co‐expression with SialDrGalT and SialTnFUT9a in ΔXT/FT *N. benthamiana* plants. The presence of Lewis X on Oo‐ASP‐1 was confirmed with MALDI‐TOF MS (Figure 6a). Expression of SialDrGalT (driven by the GPAII promoter) resulted predominantly in mono‐antennary N‐glycans. To achieve diantennary N‐glycans, we additionally co‐expressed *Homo sapiens N*‐acetyl‐glucosaminyltransferase II (HsGnTII), which allowed for the synthesis of diantennary Lewis X glycans (Figure 6b).

After generating Lewis X N‐glycans on Oo‐ASP‐1, we evaluated whether fucosidase isoform 3066 from *Emticicia oligotrophica* could remove antennary fucose from Lewis X to yield galactosylated N‐glycans. To this end, a 6xHis‐tagged variant of fucosidase isoform 3066 was produced in *E. coli* and purified from the lysate supernatant by Ni‐NTA chromatography. SDS‐PAGE confirmed the production and purity of the fucosidase, showing a prominent ~50 kDa band (Figure S9). Fucosidase 3066 was then used to remove antennary α1,3‐fucose from the Oo‐ASP‐1 N‐glycans, and the efficiency of defucosylation was confirmed by MALDI‐TOF‐MS (Figure 6a–d). This demonstrated that galactose‐extended N‐glycans could be engineered efficiently via the Lewis X engineering strategy. Showing the versatility of this strategy, we observed that similar levels of galactosylation were obtained when engineering SmKappa‐5 (Figure S10). The Lewis X strategy was found also to be compatible with engineering core α1,6‐fucosylation with DmFUT8, as fucosidase 3066 could not cleave core α1,6‐fucose (Figure S11). Taken together, we have shown that the Lewis X strategy is highly versatile, yielding high levels of galactosylation on glycoproteins produced in *N. benthamiana*.

## Discussion

Achieving efficient galactosylation of N‐glycans is challenging in plant‐based expression platforms such as *Oryza sativa* (rice) suspension cells (Kang *et al*., 2015), *Physcomitrium patens* (moss) (Bohlender *et al*., 2020) and *Nicotiana tabacum* BY‐2 cells (Navarre *et al*., 2017). Problematic galactosylation has also been reported for a variety of glycoproteins produced in *N. benthamiana*, including erythropoietin, transferrin, α1‐antitrypsin, and Oo‐ASP‐1 (Kriechbaum *et al*., 2020; Zwanenburg *et al*., 2023). The first endogenous β‐galactosidase to be characterized in *N. benthamiana* was shown to contribute to the cleavage of β1,4‐linked galactose from different glycans (Kriechbaum *et al*., 2020). Unfortunately, the removal of NbBGAL8 by CRISPR did not result in near complete galactosylation, indicating the presence of other β‐galactosidases that hamper efficient galactosylation in *N. benthamiana*. Based on this, we set out to identify the remaining NbBGALs that are able to trim terminal galactoses from engineered glycans.

Our bioinformatic analysis identified 15 different NbBGALs, which were expressed in leaf tissue or up‐regulated after *Agrobacterium*‐mediated transformation. A selection of these enzymes was made for further analysis based on their expression levels, previous proteomic studies and their predicted protein domains. We confirmed these selected NbBGALs (excluding NbBGAL16A) to localize into the apoplastic space, where they could process galactosylated glycan structures. Although we identified several NbBGALs potentially capable of modifying mammalian‐type glycans, some NbBGALs might have been missed during our analysis. The ploidy level of *N. benthamiana* complicates the assembly of a complete genome, which might lead to an incomplete overview of all NbBGAL sequences (Fernandez‐Pozo *et al*., 2015; Ranawaka *et al*., 2023). Furthermore, the recent duplication of the genome that resulted in the current ploidy caused many expressed genes to lose their function, as previously observed during the identification of active β‐hexosaminidases in *N. benthamiana* (Alvisi *et al*., 2021). Also, the spatiotemporal expression during leaf development and protein turnover during growth and development might influence the availability of glycan‐modifying NbBGALs. Nevertheless, by focusing on highly expressed NbBGALs in leaf tissue and elevated expression after *Agrobacterium*‐mediated transformation, we likely identified the key contributors to glycan modification of plant‐produced glycoproteins. Supporting this, our analysis identified NbBGAL8, previously shown to be a major contributor to the removal of terminal galactoses on mammalian‐like N and O‐glycosylated proteins in the apoplastic space (Kriechbaum *et al*., 2020). NbBGAL3B, which showed the highest expression of our selected NbBGALs under our selection criteria, was also able to modify terminal galactoses on mammalian‐like N‐glycans *in vivo*. Besides NbBGAL3B and NbBGAL8, we observed some weak *in vivo* and *in vitro* activity for NbBGAL10 and considerable activity for a mCitrine‐tagged version of NbBGAL17.

This prompts the question of how the overexpressed NbBGALs mimic native conditions, particularly in terms of cellular localization and enzymatic activity. The CaMV35S promoter drives expression approximately 10–50 times higher than that of most housekeeping genes in *N. benthamiana* (Shakhova *et al*., 2022) and has previously been associated with altered localization or atypical distribution of overexpressed proteins in plants (Groves *et al*., 2019). The subset of NbBGALs selected in this study all have a predicted signal peptide and an apoplastic localization (Figures 1 and 2). Previously, four NbBGALs have been found in apoplastic extracts after *Agrobacterium*‐mediated transformation (Grosse‐Holz *et al*., 2018), which is in line with our localization results. This suggests that our localization data closely replicated the native localization conditions. Surprisingly, the NbBGAL17‐mCitrine fusion showed a rather unusual localization pattern, which might be an artefact of the 35S‐driven overexpression. Confocal microscopy results showed an intracellular signal resembling ER and Golgi localization, suggesting that the majority of the protein was either inefficiently secreted or has undergone rapid turnover in the apoplastic space. Since NbBGAL17‐mCitrine was extractable from the apoplastic space, where it also has been found previously (Grosse‐Holz *et al*., 2018), we conclude that this particular isoform is secreted into the apoplastic space. Similarly, both confocal and western blot data revealed only an intracellular signal for NbBGAL16A‐mCitrine, indicating an intracellular localization.

In this study, we tested the selected NbBGALs *in vivo* on both mammalian‐like O‐ and N‐glycan substrates. During the initial characterization of NbBGAL8, the ability to cleave β1,3‐galactose from core 1 O‐glycans was tested *in vitro* (Kriechbaum *et al*., 2020). NbBGAL8 was able to reduce the amount of core 1 O‐glycan from 60% to 40% in 1 h and to 14% after a prolonged incubation of 4 h, indicating the potential of NbBGALs to cleave β1,3‐galactoses. However, our *in vivo* lectin‐based screenings did not reveal any other candidate that could cleave β1,3‐galactoses upon overexpression. The lack of other NbBGALs cleaving β1,3‐galactose was in accordance with the high amount of core 1 O‐glycans obtained and thus with the lack of glycosidic hydrolases trimming this structure (Castilho *et al*., 2012). While the screened NbBGALs were not able to cleave β1,3 galactoses from O‐glycans, the same might not be true for β1,4‐linked galactose residues on extended O‐glycans. The synthesis of these extended O‐glycans has not yet been attempted in a plant‐based expression system, but the presence of endogenous BGALs might hamper these efforts in the future.

While screening for beta‐galactosidase activity against N‐glycans, we identified two NbBGALs, namely NbBGAL8 and NbBGAL3B, as the most efficient enzymes able to cleave β1,4‐linked galactose from engineered N‐glycans. MALDI‐TOF‐MS showed that degalactosylation by NbBGAL8 was near complete, while overexpression of NbBGAL3B resulted in cleavage of approximately half of the galactose residues. However, as discussed before, overexpression of the NbBGALs was not representative of the natural expression pattern. NbBGAL3B and NbBGAL8 were selected based on their high upregulation after agroinfiltration. Interestingly, NbBGAL3B expression was approximately three times higher than NbBGAL8. Overall, our data indicate that NbBGAL3B and NbBGAL8 collectively drive degalactosylation in *N. benthamiana*.

To complement the *in viv*o data, we performed an *in vitro* screening of extractable NbBGAL‐mCitrine fusions. We first tested our NbBGAL extracts on galactobiose substrates with different linkages, which has previously been performed for recombinant versions of radish (Kotake *et al*., 2005), tomato (Ishimaru *et al*., 2009) and *Arabidopsis* (Gantulga *et al*., 2008, 2009) β‐galactosidases (Table S4). Interestingly, both RsBGAL1 and TBG5 showed a similar preference for β1,3/β1,6‐linkages as their NbBGAL8 orthologue, while TBG4 (BGAL2 orthologue), AtBGAL2 and NbBGAL2A preferred β1,3/ β1,4‐galactose linkages. These data might indicate that enzymatic activity is (partially) conserved between different plant species. In contrast, we were not able to show the activity of NbBGAL3B and NbBGAL17 on these substrates, while NbBGAL10 showed only a mild activity on β1,3‐galactobiose.

The NbBGAL‐mCitrine extracts were further tested on *in vitro* galactosylated SmKappa‐5. Both NbBGAL8 and NbBGAL17 were able to modify these N‐glycans *in vitro*, whereas NbBGAL3B did not show any activity. Interestingly, NbBGAL17 showed activity against an N‐glycan substrate, while it did not show any activity on the galactobiose substrates. Every NbBGAL has a predicted N‐terminal galactose binding domain, and some have a galectin domain (Figure 1d). It has been hypothesized that these domains are responsible for anchoring the enzymes to specific substrates and thereby determine their specificity (Ahn *et al*., 2007). Both mCitrine‐tagged NbBGAL8 and NbBGAL17 were truncated, ~45‐kDa versions of these enzymes that plausibly lack the galactose binding domain and galectin domain. We hypothesize that these truncations influence their substrate specificity. This possibility was previously discussed by Kriechbaum *et al*. (2020), who also suggested that the truncated version represents an active form of the enzyme. Similarly, RsBGAL1 exhibited truncated versions, without binding domains, suggesting that truncation is a naturally occurring phenomenon of eventual functional significance (Kotake *et al*., 2005). These truncations may affect their enzymatic specificity, potentially explaining their ability to modify N‐glycan substrates. NbBGAL2A did not show the same truncation pattern as NbBGAL8 and NbBGAL17, while having a high preference for β1,4‐galactose linkages and not being able to hydrolyse galactosylated N‐glycans on SmKappa‐5. However, overexpression of the native NbBGAL17 did not result in detectable cleavage of galactose from engineered N‐glycans, supporting the idea that NbBGAL8 and NbBGAL3B are primarily responsible for the undesired galactosidase activity.

As no activity was detected for NbBGAL3B *in vitro*, we sought to confirm that the mCitrine tag did not affect its activity. We repeated the *in vivo* experiment with mCitrine‐tagged NbBGALs and showed that the mCitrine‐tagged NbBGAL3B, NbBGAL8 and also NbBGAL17 can modify terminal β1,4‐galactosylated N‐glycans, thereby indicating that the tag did not interfere with the *in vivo* activity of NbBGAL3B. Substantial amounts of truncated NbBGAL17‐mCitrine were observed in apoplastic extracts, which likely accounted for the *in vivo* activity and contrasted starkly with the results from the untagged NbBGAL17. The lack of activity of NbBGAL3B under *in vitro* conditions was surprising, suggesting either that the conditions for enzymatic activity were not met or that an inactive form was purified. A recombinantly produced orthologue from Arabidopsis (AtBGAL3) exhibited activity *in vitro* under conditions like those used in this study (Gantulga *et al*., 2009). Furthermore, there are no known co‐factors for the GH35 family, which suggests that we extracted an inactive form of the enzyme. Our data showed that NbBGALs might be able to form dimers (Figure 1c,d). We were able to detect a signal, corresponding to approximately twice the original size of the tagged versions of NbBGALs in our western blots, even under denaturing conditions. Dimers have been observed in chickpea (Kishore and Kayastha, 2012) and archaea (Kil *et al*., 2024) GH35 members, while an *E. coli* β‐galactosidase was even able to form a tetramer that was crucial for its activity (Jacobson *et al*., 1994). We might not have been able to replicate correct dimerization under our *in vitro* conditions, hence affecting the activity of NbBGAL3B.

During β1,4‐galactosidase activity screenings on N‐glycans, we observed unexpected peaks with masses corresponding to N‐glycans containing additional pentose and hexose residues. We detected up to five additional non‐engineered pentose residues and four hexose residues. The addition of non‐engineered pentoses is rare but has been reported in *P. patens* (Bohlender *et al*., 2020, 2022) and *N. tabacum* (Kittur *et al*., 2020). In *P. patens*, up to five additional (methylated) pentoses have been reported, whereas in *N. tabacum* up to three additional non‐methylated pentoses were detected. The pentose residues in *P. patens* were confirmed to be arabinose residues (Bohlender *et al*., 2022). Interestingly, no additional hexose residues were reported in *P. patens*, which contrasts with the previous report in *N. tabacum* and our findings. In *N. tabacum*, N‐glycans with an additional hexose were detected, but the identity of both pentose and hexose residues remains unknown (Kittur *et al*., 2020). Our enzymatic digestion identified the pentose and hexose residues as arabinofuranose and galactose, respectively, but the exact structure and linkages remain unknown. The difference observed in residues suggests that different structures are synthesized in *Nicotiana* species compared to *P. patens*. Possibly, arabinan chains are synthesized on N‐glycans from *P. patens* and galactan chains on N‐glycans from *Nicotiana* species. We expect that aberrant localization of the SialDrGalT, caused by the sialyltransferase CTS domain, induced non‐stringent cis/medial Golgi localization instead of the intended trans‐Golgi localization (McGinness *et al*., 2024). Improper localization might cause the galactosylation to be catalysed in the improper Golgi compartment, leading to the observed N‐glycan structures with non‐engineered arabinofuranoses and galactoses. Providing DrGalT with a stringent CTS domain might solve this issue and simultaneously improve galactosylation.

To produce recombinant glycoproteins with galactosylated glycans without an available NbBGAL KO line, we devised a novel Lewis X strategy. This strategy resembles the protective capacity of sialic acid capping to shield galactose residues and subsequent removal to obtain complete galactosylation (Kriechbaum *et al*., 2020). The sialic acid capping strategy depends on introducing or stably transforming the biosynthetic pathway for the nucleotide sugar and sialyltransferase to attach sialic acid to galactose into the plant (Castilho *et al*., 2010). In contrast, the Lewis X strategy presented in this work only depends on introducing a single fucosyltransferase to create the Lewis X motif as the donor nucleotide sugar is already present in the proper Golgi compartment. After purifying the glycoprotein, selective removal of the α1,3‐linked fucose residue from the Lewis X motif by fucosidase treatment yields a galactosylated N‐glycan. The level of galactosylation on N‐glycans could be drastically increased through this method, as suggested by the large increase observed for the galactosidase‐prone Oo‐ASP‐1 protein. Interestingly, this strategy also hinders the synthesis of the unusual arabinose and galactose residues, observed when engineering terminal β1,4‐galactoses on our vaccine candidates. Furthermore, this method enables the selection of mono‐ or diantennary galactosylated N‐glycans and has the potential to efficiently generate tri‐ or tetraantennary galactosylated N‐glycans through the expression of GnTIV and/or GnTV (Castilho *et al*., 2011). The Lewis X strategy will enable efficient synthesis of biopharmaceuticals and helminth vaccines with terminal galactoses on N‐glycans.

In conclusion, we revealed that in addition to the previously characterized NbBGAL8 (Kriechbaum *et al*., 2020), the highly expressed isoform NbBGAL3B can cleave β1,4‐linked galactose on engineered N‐glycans in *N. benthamiana*. We also showed that NbBGAL17 has the potential to cleave terminal β1,4‐linked galactose on N‐glycans, but only after reaching the apoplast with a mCitrine tag. In contrast, β1,3‐linked galactose on O‐glycans was not cleaved by any of our BGAL candidate enzymes, besides the already characterized NbBGAL8. We devised a strategy to protect the terminal β1,4‐linked galactose on N‐glycans by using naturally occurring steric hindrance of the NbBGAL enzymes. By synthesizing a Lewis X structure on our helminth glycoproteins, we showed that the terminal galactose could be protected *in vivo*. By removing the antennary fucose *in vitro* with a bacterial α1,3‐fucosidase, we were able to synthesize relatively homogeneous terminal galactosylated glycoproteins. During the identification of the β‐galactosidases, the presence of additional arabinofuranose and galactose residues on N‐glycans was discovered as well, which was largely solved by using the Lewis X strategy. This unusual arabinosylation and galactosylation will have to be addressed in the future to optimize biopharmaceutical glycoprotein production in *N. benthamiana*. The identification of problematic β‐galactosidases in this study lays the groundwork for NbBGAL8 and NbBGAL3B KO lines to achieve near complete galactosylation on recombinant glycoproteins. Our novel Lewis X strategy for the protection of galactose residues from β‐galactosidases could be used to bridge the time gap until such knockout lines are available.

## Materials and methods

### Identification and bioinformatic analysis of *N. benthamiana* BGAL orthologues

To generate an inventory of the different *N. benthamiana* BGALs, we blasted the *A. thaliana* BGAL (AtBGAL) amino acid sequences (BGAL1‐17 from TAIR database) against the *N. benthamiana* draft genomes from the Sol Genomics Network (version Niben1.0.1) (Fernandez‐Pozo *et al*., 2015) and Benthgenome (www.benthgenome.com v6.1) (Ranawaka *et al*., 2023). The identified genes were then compared between the SOL genomics and Benthgenome databases and named according to the nomenclature and orthology to AtBGALs. Expression data of the BGALs in different tissues were obtained from Benthgenome, and mock‐infiltration expression data were obtained from published literature (Buscaill *et al*., 2019). The phylogenetic tree was constructed by first aligning the amino acid sequences by ClustalW using standard settings. A phylogenetic tree was constructed using the maximum likelihood method of MEGA‐X in default mode with a bootstrap test of 1000 replicates. Heatmaps were generated using R (version 4.4.2) with the packages RColorBrewer and gplots. NbBGAL localization based on proteomics data was extracted from literature (Grosse‐Holz *et al*., 2018). Protein domains of the identified BGALs were determined by uploading the amino acid sequences into the InterPro search engine (Paysan‐Lafosse *et al*., 2023).

### Cloning of NbBGAL constructs for protein expression and subcellular localization

cDNA was prepared from *N. benthamiana* leaf RNA by first using the Maxwell® 16 LEV Plant RNA Kit (Promega; AS1430) followed by the Maxima H Minus First Strand cDNA Synthesis Kit (Thermo Scientific™, K1681). The open reading frames of the selected NbBGALs were amplified using Phusion®High‐Fidelity Polymerase (New England Biolabs; M0530S) with appropriate primers (Table S1) that contained flanking restriction sites for subcloning. Amplified NbBGALs were cloned into pCR2.1‐TOPO using a TOPO™ TA Cloning™ Kit (Thermo Fisher, 450641), transformed into *E. coli* TOP10 cells (Thermo Fisher; C404052) and verified by colony PCR. Positive colonies were miniprepped and the plasmids were sent for Sanger sequencing (Macrogen, Netherlands). Verified NbBGAL sequences were further cloned into the pHYG plant expression vector using standard digestion and ligation procedures (Westerhof *et al*., 2012).

To confirm the subcellular localization of the NbBGALs, mCitrine was fused to the NbBGALs through Gibson assembly. In addition to adding mCitrine, a 6xHIS tag and a strep‐tag were included. The NbBGAL open reading frames were reamplified with appropriate primers (Table S2) using Phusion DNA polymerase (New England Biolabs; M0530S). The newly constructed open reading frames were cloned into the pRAP vector (Wilbers *et al*., 2017) via Gibson assembly using the NEBuilder HiFi DNA Assembly Master Mix (New England Biolabs; E2621S). After verifying the sequence, the expression cassette was transferred into pHYG as previously described (Westerhof *et al*., 2012).

### Other expression vectors

In addition to the cloned NbBGALs, the following expression vectors were used to complement this study: (1) P19 silencing repressor (Garabagi *et al*., 2012), (2) the *Danio rerio* β1,4‐galactosyltransferase fused to the CTS region of the rat a2,6‐sialyltransferase under control of the GPAII promoter (GPAII:SialDrGalT) (Wilbers *et al*., 2017), (3) SialDrGalT under control of the dual 35S promoter (35S:SialDrGalT) (Wilbers *et al*., 2017), (4) *Ostertagia ostertagi* activation‐associated secreted protein 1 (Oo‐ASP‐1) (Zwanenburg *et al*., 2023), (5) *Schistosoma mansoni Kappa‐5* (Wilbers *et al*., 2017), (6) *Drosophila melanogaster* fucosyltransferase 8 (DmFut8) (Wilbers *et al*., 2016), (7) *Schistosoma mansoni* fucosyltransferase C (SmFucTC) (van Noort *et al*., 2020), (8) a hybrid *Tetraodon nigriviridus* α1,3‐fucosyltransferase IXa (SialTnFut9a) (Wilbers *et al*., 2017) and (9) *Drosophila melanogaster* core 1 β1,3‐galactosyltransferase (C1GalT1) (Castilho *et al*., 2012). Furthermore, two additional constructs were created: (1) *Cooperia oncophora* double‐domain activation‐associated secreted protein (dd‐Co‐ASP) and (2) *Homo sapiens* polypeptide N‐acetylgalactosaminetransferase 1 (HsGalNT1). Both genes were ordered at IDT and flanked by the NheI/KpnI and NcoI/KpnI restriction sites, respectively, for cloning into the pHYG vector. Oo‐ASP‐1, SmKappa‐5 and dd‐Co‐ASP were preceded by a chitinase signal peptide and N‐terminal 6× histidine and FLAG tags.

### Agroinfiltration of *Nicotiana benthamiana* leaves

*Agrobacterium tumefaciens* (strain MOG101) culturing was done as described previously (Wilbers *et al*., 2017). Bacteria were resuspended to an optical density (OD) of 0.5. When co‐infiltrating with DmFUT8, the OD was adjusted to 0.1. The youngest fully expanded leaves of 5–6‐week‐old ΔXT/FT (Strasser *et al*., 2008) or wild‐type *N. benthamiana* plants were infiltrated. Infiltrated leaves were harvested after 3 or 5 days post‐infiltration for subcellular localization or protein expression, respectively.

### Subcellular localization

For subcellular localization studies, leaves of WT *N. benthamiana* plants were infiltrated with Agrobacterium clones carrying the N‐terminal mCitrine‐tagged NbBGALs and mCherry‐tagged Formin as a plasma membrane marker (Favery *et al*., 2004). After 3 days, the leaves were harvested and incubated in ½ MS medium supplemented with 500 mM NaCl for 5–15 min to induce plasmolysis. The images were acquired using a Stellaris 5 Confocal LSM (Leica). mCitrine was excited at 513 nm and detected at 530 nm. mCherry was excited at 587 nm and detected at 630 nm. Images were processed using the Leica Las X office software post‐acquisition.

### Protein isolation and purification

Infiltrated leaves were harvested and afterwards processed to obtain apoplast proteins and intracellular proteins (crude extract), as previously described (Alvisi *et al*., 2021). Recombinant glycoproteins were purified from the apoplast using HisPur Ni‐NTA resin (Thermo Fisher; 88222). After elution, purified proteins were dialysed to 1× PBS. Protein concentrations were determined using a Pierce™ BCA protein assay kit (Thermo Scientific; 23225).

### SDS‐PAGE and western blots

Apoplastic proteins and intracellular fractions containing the BGAL‐mCitrine fusions were analysed using western blot and protein staining. Proteins were run on 12% (w/v) mPAGE gels (MerckMillipore; MP12W15) under reducing conditions. The gels were either stained with Coomassie Brilliant Blue R250 (CBB) or transferred to a PVDF membrane using the Turbo transfer blotter (BioRad) for use in western blot. PVDF membranes were further developed with an anti‐GFP antibody as described previously (Alvisi *et al*., 2021).

### Glycan analysis

To screen for the presence of galactosylated glycans, lectin‐binding assays were performed as previously described (Alvisi *et al*., 2021). Screening for terminal β1,4‐linked galactose on N‐glycans of purified SmKappa‐5 was done with the biotinylated Ricinus Communis Agglutinin I (RCA‐I; Vector Biolabs; B‐1085‐1). Screening for the absence/removal of β1,3‐linked galactose on O‐glycans of dd‐Co‐ASP was performed with the Vicia Villosa Lectin (VVL, VVA; Vector Biolabs; B‐1235‐2) and Peanut Agglutinin (PNA; Vector Biolabs; B‐1075‐5) according to the same protocol. VVA binds to α‐ or β‐linked terminal *N*‐acetylgalactosamine, and PNA binds to galactosyl(β‐1,3)*N*‐acetyl‐galactosamine O‐glycan (T‐antigen). Plates were coated with proteins at a concentration of 10 μg/mL. Biotinylated lectins were incubated at a concentration of 5 μg/mL for RCA‐I, 2 μg/mL for VVA and 5 μg/mL for PNA.

To qualitatively determine the composition of the N‐glycans, a mass spectrometry analysis was performed. The N‐glycans of purified glycoproteins were released and purified as previously described (Wilbers *et al*., 2017). Released N‐glycans were analysed by matrix‐associated laser desorption/ionization time‐of‐flight mass spectrometry (MALDI‐TOF‐MS). MS spectra were obtained using an Autoflex Max MALDI‐TOF‐MS (Bruker Daltonics) in positive‐ion reflection mode for unlabelled glycans and negative‐ion reflection mode for labelled glycans. Samples undergoing an enzyme treatment were labelled beforehand as previously described (Wilbers *et al*., 2017). After labelling, they were treated with β‐*N*‐acetyl‐glucosaminidase from *Streptococcus pneumoniae* (NEB; P0744S) to verify the presence of galactose‐extended antenna. After enzyme treatment, the samples were cleaned up using a C18 ZipTip™ (MerckMillipore; ZTC18S096) and then assessed again using MALDI‐TOF‐MS. To identify unknown N‐glycan residues, labelled N‐glycans were treated with either 0.075 U α‐L‐Arabinofuranosidase (*Aspergillus niger*) (Megazyme; 700004179) or 1 U of β‐Galactosidase (*Aspergillus niger*) (Megazyme; 700004192) or with both enzymes. The labelled N‐glycan solution (1 μL) was mixed with the enzyme(s), 100 mM sodium acetate buffer (pH 4.5) at a final volume of 10 μL and incubated at 40°C for 1 h under agitation. Post‐incubation, samples were cleaned up using a C18 ZipTip™ and measured on the MALDI‐TOF‐MS.

### α1,3‐fucosidase and B4GALT expression in *E. coli*

A plasmid harbouring the open reading frame of fucosidase isoform Eo3066 of *Emticicia oligotrophica* was kindly provided by Liu *et al*. (2016). The open reading frame was cloned into the pCold expression vector (TakaraBio, Japan; 3360) via Gibson assembly using NEBuilder HiFi DNA Assembly Master Mix (New England Biolabs; E2621S). When synthesizing PCR products with Phusion DNA polymerase (New England Biolabs; M0530S) for Gibson assembly, a 6xHis tag was introduced at the N‐terminus by PCR using the appropriate primers (Table S3). The resulting pCold expression vector harbouring the fucosidase was finally transformed into *E. coli* BL21 competent cells (TaKaRa, Japan; 9126).

An *E. coli* (strain K12) codon‐optimized sequence of *Mus musculus* (mouse) beta‐1,4‐galactosyltransferase 1 (Uniprot P15535, Δ44‐399) was ordered at IDT. The fragment was cloned into the previously mentioned pCold vector, in between the translation enhancing element (TEE) and a C‐terminal 6× histidine followed by a maltose‐binding protein (MBP) tag using the appropriate primers (Table S3).

Fucosidase and beta‐1,4‐galactosyltransferase 1 were expressed and purified as follows. BL21 cells harbouring the expression vectors were grown to an optical density (OD) 600 of 0.6–0.8 at 37 °C in Lysogeny broth (LB) medium, followed by an incubation on ice for 30 min. Expression was induced with 0.5 mM IPTG and the cells were grown at 15 °C for 16–24 h while shaking at 200 rpm. The cells were spun down and redissolved in ice‐cold extraction buffer (20 mM HEPES, 300 mM NaCl, 25 mM Imidazole at pH 7.4). The cells were disrupted with glass beads (500–750 μm, Thermo Scientific Chemicals, 397641000) in a bead mill at 30 m/s for 8 min. The disrupted cells were spun down at full speed at 4 °C for 5 min and the supernatant was allowed to bind to pre‐equilibrated HisPur™ Ni‐NTA Resin (Thermo Scientific). The resin was washed 3 times with 4 volumes of ice‐cold extraction buffer and eluted with 1 volume of elution buffer (20 mM HEPES, 300 mM NaCl, 250 mM Imidazole at pH7.4). Purity of the produced proteins was analysed by SDS‐PAGE gel stained by Coomassie Brilliant Blue. The eluted fucosidase was exchanged to 50 mM phosphate–citrate buffer (pH 6.6) and the B4GALT enzyme was exchanged to 25 mM HEPES, 100 mM NaCl (pH6.8) using Slide‐A‐Lyzer mini dialysis cups (Thermo Fisher; 69552).

### *In vitro* glycan engineering

Terminal antennary fucose of Lewis X‐engineered N‐glycans on purified glycoproteins was removed with fucosidase Eo3066 (1:20 enzyme to substrate ratio) in 50 mM phosphate–citrate buffer (pH 6.6), followed by incubation for 2 h at 37 °C. The absence of antennary fucose was further analysed by MALDI‐TOF MS.

Terminal galactose residues were added to the N‐glycans of SmKappa‐5 as follows. Purified SmKappa‐5 was exchanged to 25 mM HEPES, 100 mM NaCl (pH6.8) using Slide‐A‐Lyzer mini dialysis cups (Thermo Fisher; 69552). SmKappa‐5 was then incubated with the purified B4GALT enzyme (1:20 enzyme to substrate ratio), 500 μM UDP‐galactose (Merck, 670111‐M) and 50 mM HEPES pH 6.8, while shaking at 37 °C for 2 h. Addition of the terminal galactose was determined by MALDI‐TOF MS analysis.

### *In vitro* β‐galactosidase activity assay

Constructs harbouring the untagged NbBGALs and mCitrine‐tagged NbBGALs were expressed in wild‐type *N. benthamiana* as previously described and harvested 5 days post‐infiltration. 10 μL of the apoplastic extract was first analysed on SDS‐PAGE gels and by western blotting with anti‐GFP antibodies for GFP detection as previously described.

The P19 (control) and NbBGAL extracts were tested on β1,3‐galactobiose (carbosynth, OG10186), β1,4‐galactobiose (megazyme, O‐GBI4‐20MG) and β1,6‐galactobiose (megazyme, O‐GBI6). 10 μL apoplastic extract was mixed with 30 μg galactobiose, 50 mM sodium acetate buffer (pH 4.8) at a final volume of 20 μL for 1 h while shaking at 37 °C. Half of the mix was loaded and visualized by thin‐layer chromatography (TLC) as described in (Nibbering *et al*., 2020).

For the β‐galactosidase activity assay on glycoproteins, SmKappa‐5 with diantennary galactosylated N‐glycans was first buffer exchanged to 25 mM Sodium acetate (pH 4.8) as previously described. 25 μL apoplastic extract was mixed with 100 μg *in vitro* glyco‐engineered SmKappa‐5, 50 mM sodium acetate buffer (pH 4.8) at a final volume of 50 μL for 2 h while shaking at 37 °C. The reaction products were analysed on MALDI‐TOF‐MS.

## Author contributions

AK, MJMB, LN, PN, SM and HO designed and performed the experiments, advised by PN and RHPW. AK, MJMB, LN, RHPW and PN analysed the data. PN and RHPW conceived the project. AK and PN wrote the draft manuscript that was edited by AS and RHPW. RHPW and AS provided funding. All authors read and approved the final article.

## Funding

This project has received funding from the International Coordination of Research on Infectious Animal Diseases (ICRAD; Plant4Nemavax project), the Netherlands Organisation for Scientific Research Grant 16740 from Netherlands Organization for Scientific Research, The European innovation programme, Eurostars (E! 115017). This work was supported by a grant from the centre for unusual collaboration (an alliance between Eindhoven University of Technology, Utrecht University, the University Medical Center Utrecht and Wageningen University and Research).

## Conflict of interest

The authors declare that the research was conducted without any commercial or financial relationships that could be construed as a potential conflict of interest.

## Supporting information


**Figure S1** Localization of mCitrine‐tagged NbBGALs. Confocal laser scanning microscope images of leaf epidermal cells transiently expressing different NbBGAL‐mCitrine and the plasma membrane marker formin‐mCherry. Leaves were treated with 500 mM NaCl to induce plasmolysis and to allow the plasma membrane to collapse inside the cell. The mCitrine and mCherry signals were captured using a Stellaris 5 Confocal LSM (Leica). Scalebar is 10 μm.
**Figure S2** Cleavage of terminal β1,4‐linked galactose from N‐glycan by NbBGALs. SmKappa‐5 was co‐expressed with 35S:SialDrGalT and different NbBGALs in ΔXT/FT *N. benthamiana* plants. MALDI‐TOF‐MS N‐glycan analysis of purified SmKappa‐5 upon co‐expression of 35S:SialDrGalT with NbBGAL1B (a), NbBGAL2A (b), NbBGAL3A (c), NbBGAL8‐like (d), NbBGAL9A (e), NbBGAL10 (f), NbBGAL16A (g) or NbBGAL17 (h). All samples were treated with β‐N‐acetyl‐glucosaminidase to confirm the presence of galactose‐extended antenna. Peaks of interest were labelled with the corresponding N‐glycan structures.
**Figure S3** Enzymatic digestion of β1,4‐galactose engineered N‐glycans containing unknown glycan residues. To validate the presence and identity of the additional hexose and pentose residues, the N‐glycans were treated with *Aspergillus niger* α‐L‐arabinofuranose and/or β‐galactosidase and analysed by MALDI‐TOF‐MS. MS profiles are given for SmKappa‐5 upon co‐expression of 35S:SialDrGalT without enzymatic treatment of the N‐glycans (a), or after treatment with α‐L‐arabinofuranose (b), β‐galactosidase (c) or both (d). A table representing the different structures (1–34) is presented as well (e).
**Figure S4** Enzymatic activity of NbGAL‐mCitrine‐tagged enzymes against β1,3/β1,4/β1,6‐linked galactobiose. Apoplast fluids from a P19 control infiltration or a selection of overexpressed NbBGAL‐mCitrine fusions were incubated with galactobiose at pH 4.8 in sodium acetate buffer. Enzymatic activity was visualized with thin‐layer chromatography versus a galactose and associated undigested galactobiose control.
**Figure S5** *In vitro* galactosylation of SmKappa‐5 by MmGALT‐6xHIS‐MBP. (a) SmKappa‐5 was transiently overexpressed in ΔXT/FT *Nicotiana benthamiana* plants and purified from the apoplastic fluid via Ni‐NTA Purification. (b) Codon‐optimized *Mus musculus* beta‐1,4‐galactosyltransferase 1 was expressed in the *E. coli* BL21 line, tagged with an N‐terminal 6xhistidine and maltose‐binding protein (MBP) for enhanced solubility. MmGALT‐6xHIS‐MBP was purified from *E. coli* via Ni‐NTA Purification. (c) Purified SmKappa‐5 was galactosylated *in vitro* with MmGALT‐6xHIS‐MBP. N‐glycans were released with PNGase F and analysed with MALDI‐TOF. The presented profiles show that SmKappa‐5 is fully galactosylated after the enzymatic reaction.
**Figure S6** *In vitro* β‐galactosidase activity of NbBGALs. MALDI‐TOF‐MS N‐glycan analysis of *in vitro* glyco‐engineered SmKappa‐5 after treatment with apoplast fluids from p19 control infiltrations or apoplast fluids upon overexpression of NbBGAL2A or NbBGAL10 (with or without mCitrine fusion).
**Figure S7** Cleavage of terminal β1,4‐linked galactose from N‐glycan by NbBGAL‐mCitrine fusions. SmKappa‐5 was co‐expressed with 35S:SialDrGalT and different NbBGALs fusion proteins in ΔXT/FT *N. benthamiana* plants. MALDI‐TOF‐MS N‐glycan analysis of purified SmKappa‐5 upon co‐expression of 35S:SialDrGalT only (a) or in combination with NbBGAL2A‐mCitrine (b), NbBGAL3B‐mCitrine (c), NbBGAL8‐mCitrine (d), NbBGAL10‐mCitrine (e) or NbBGAL17‐mCitrine (f). All samples were treated with β‐N‐acetyl‐glucosaminidase to confirm the presence of galactose‐extended antenna. Peaks of interest were labelled with the corresponding N‐glycan structures.
**Figure S8** Expression of dd‐Co‐ASP in *N. benthamiana*. SDS‐PAGE and Coomassie blue staining of apoplast fluids of dd‐Co‐ASP co‐expressed with HsGalNT1 and C1GalT. P19 was included in all samples to enhance expression. Intact dd‐Co‐ASP can be observed at ±51 kDa and cleavage products dd‐Co‐ASP can be observed at ±27 kDa. Introduction of O‐glycans on dd‐Co‐ASP with HsGalNT1 seems to partially prevent cleavage.
**Figure S9** SDS‐PAGE analysis of the production and purification of fucosidase isoform 3066 of *E. oligotrophica*.
**Figure S10** Efficient galactosylation through Lewis X engineering in *N. benthamiana* and *in vitro* removal of antennary fucose. SmKappa‐5 was co‐expressed with GPAII:sialDrGalT and SialTnFucT9a in ΔXT/FT *N. benthamiana* plants to enable Lewis X synthesis on N‐glycans of SmKappa‐5. (A‐B) MALDI‐TOF MS N‐glycan profiles of purified Oo‐ASP‐1 upon co‐expression of GPAII:SialDrGalT and SialTnFucT9a before (a) and after (b) *in vitro* fucosidase treatment. (c, d) MALDI‐TOF MS N‐glycan profiles of SmKappa‐5 upon co‐expression of GPAII:SialDrGalT, SialTnFucT9a and HsGnTII (for synthess of diantennary N‐glycans) before (c) and after (d) *in vitro* fucosidase treatment.
**Figure S11** *In vitro* fucosidase treatment of Lewis X‐engineered N‐glycans does not remove the core α1,6‐fucose on Oo‐ASP‐1 N‐glycans. Oo‐ASP‐1 was co‐expressed with GPAII:sialDrGalT and SialTnFucT9a in ΔXT/FT *N. benthamiana* plants to enable Lewis X synthesis as well as DmFUT8 for core α1,6‐fucosylation. (a, b) MALDI‐TOF MS N‐glycan profiles of Oo‐ASP‐1 upon co‐expression of SialDrGalT, TnFucT9a and DmFUT8 before (a) and after (b) *in vitro* fucosidase treatment.
**Table S1** Primers used for cloning the NbBGAL overexpression constructs.
**Table S2** Primers used for NbBGAL‐mCitrine fusion cloning.
**Table S3** Primers used for enzyme production in *E. coli*.
**Table S4** Characterized activity of recombinantly produced radish, tomato and Arabidopsis β‐galactosidases.

## Data Availability

The original contributions presented in the study are included in the article/Supplementary Materials. Further inquiries can be directed to the corresponding author.
