# Supplementary material for: Identification of β‐galactosidases along the secretory pathway of Nicotiana benthamiana that collectively hamper engineering of galactose‐extended glycans on recombinant glycoproteins
Source: Plant Biotechnol J. 2025 May 7;24(1):224–38. doi: 10.1111/pbi.70126 (PMC12854890; doi:10.1111/pbi.70126)
Supplement: Supplementary file 1 — Figure S1 Localization of mCitrine‐tagged NbBGALs. Confocal laser scanning microscope images of leaf epidermal cells transiently expressing different NbBGAL‐mCitrine and the plasma membrane marker formin‐mCherry. Leaves were treated with 500 mM NaCl to induce plasmolysis and to allow the plasma membrane to collapse inside the cell. The mCitrine and mCherry signals were captured using a Stellaris 5 Confocal LSM (Leica). Scalebar is 10 μm. Figure S2 Cleavage of terminal β1,4‐linked galactose from N‐glycan by NbBGALs. SmKappa‐5 was co‐expressed with 35S:SialDrGalT and different NbBGALs in ΔXT/FT N. benthamiana plants. MALDI‐TOF‐MS N‐glycan analysis of purified SmKappa‐5 upon co‐expression of 35S:SialDrGalT with NbBGAL1B (a), NbBGAL2A (b), NbBGAL3A (c), NbBGAL8‐like (d), NbBGAL9A (e), NbBGAL10 (f), NbBGAL16A (g) or NbBGAL17 (h). All samples were treated with β‐N‐acetyl‐glucosaminidase to confirm the presence of galactose‐extended antenna. Peaks of interest were labelled with the corresponding N‐glycan structures. Figure S3 Enzymatic digestion of β1,4‐galactose engineered N‐glycans containing unknown glycan residues. To validate the presence and identity of the additional hexose and pentose residues, the N‐glycans were treated with Aspergillus niger α‐L‐arabinofuranose and/or β‐galactosidase and analysed by MALDI‐TOF‐MS. MS profiles are given for SmKappa‐5 upon co‐expression of 35S:SialDrGalT without enzymatic treatment of the N‐glycans (a), or after treatment with α‐L‐arabinofuranose (b), β‐galactosidase (c) or both (d). A table representing the different structures (1–34) is presented as well (e). Figure S4 Enzymatic activity of NbGAL‐mCitrine‐tagged enzymes against β1,3/β1,4/β1,6‐linked galactobiose. Apoplast fluids from a P19 control infiltration or a selection of overexpressed NbBGAL‐mCitrine fusions were incubated with galactobiose at pH 4.8 in sodium acetate buffer. Enzymatic activity was visualized with thin‐layer chromatography versus a galactose and associated [file PBI-24-224-s001.pdf]

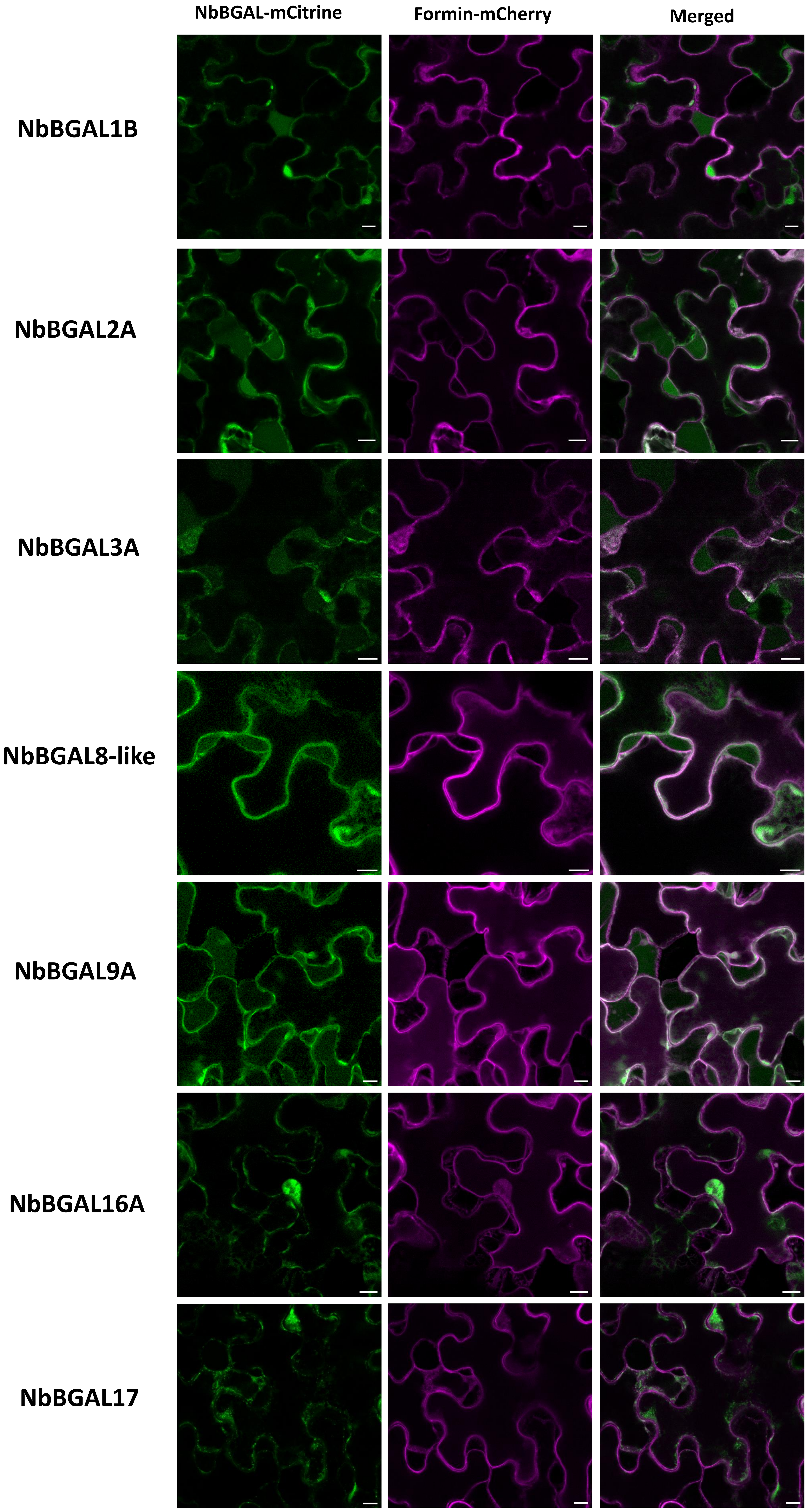

Supplemental figure 1. Localization of mCitrine tagged NbBGALs.
Confocal laser scanning microscope images of leaf epidermal cells transiently expressing different NbBGAL-mCitrine and the plasma membrane marker formin-mCherry. Leaves were treated with 500 mM NaCl to induce plasmolysis and to allow plasma membrane to collapse inside the cell. The mCitrine and mCherry signals were captured using a Stellaris 5 Confocal LSM (Leica).
Scalebar is 10 µm.

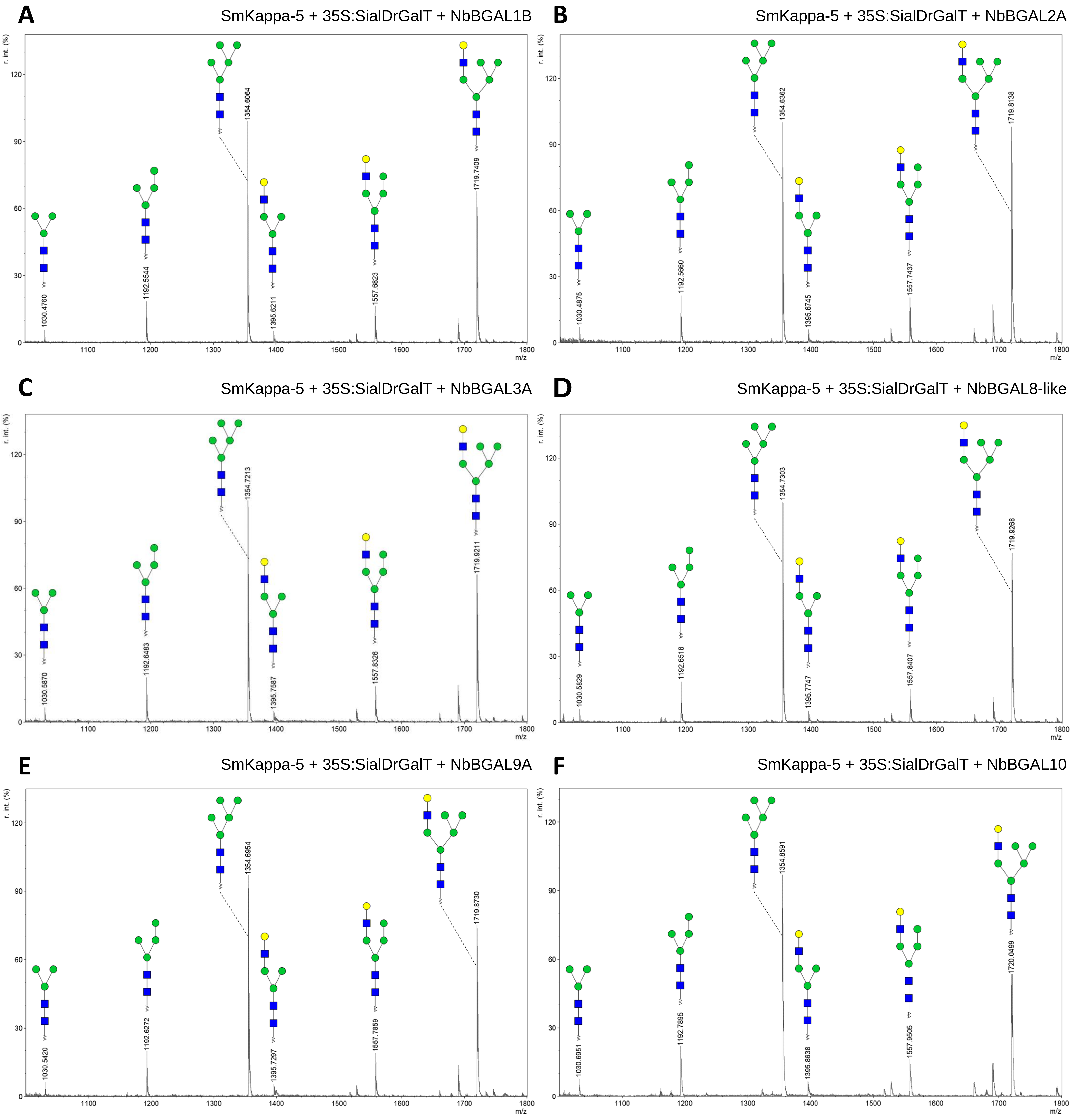

Supplemental figure 2. Cleavage of terminal β1,4-linked galactose from N-glycan by NbBGALs. SmKappa-5 was co-expressed with 35S:SialDrGalT and different NbBGALs in ΔXT/FT *N. benthmiana* plants. MALDI-TOF-MS N-glycan analysis of purified SmKappa-5 upon co-expression of 35S:SialDrGalT with NbBGAL1B **(A)**, NbBGAL2A **(B)**, NbBGAL3A **(C)**, NbBGAL8-like **(D)**, NbBGAL9A **(E)**, NbBGAL10 **(F)**, NbBGAL16A **(G)** or NbBGAL17 **(H)**. All samples were treated with β-N-acetylglucosaminidase to confirm the presence of galactose-extended antenna. Peaks of interest were labeled with the corresponding N-glycan structures.

G

SmKappa-5 + 35S:SialDrGalT + NbBGAL16A

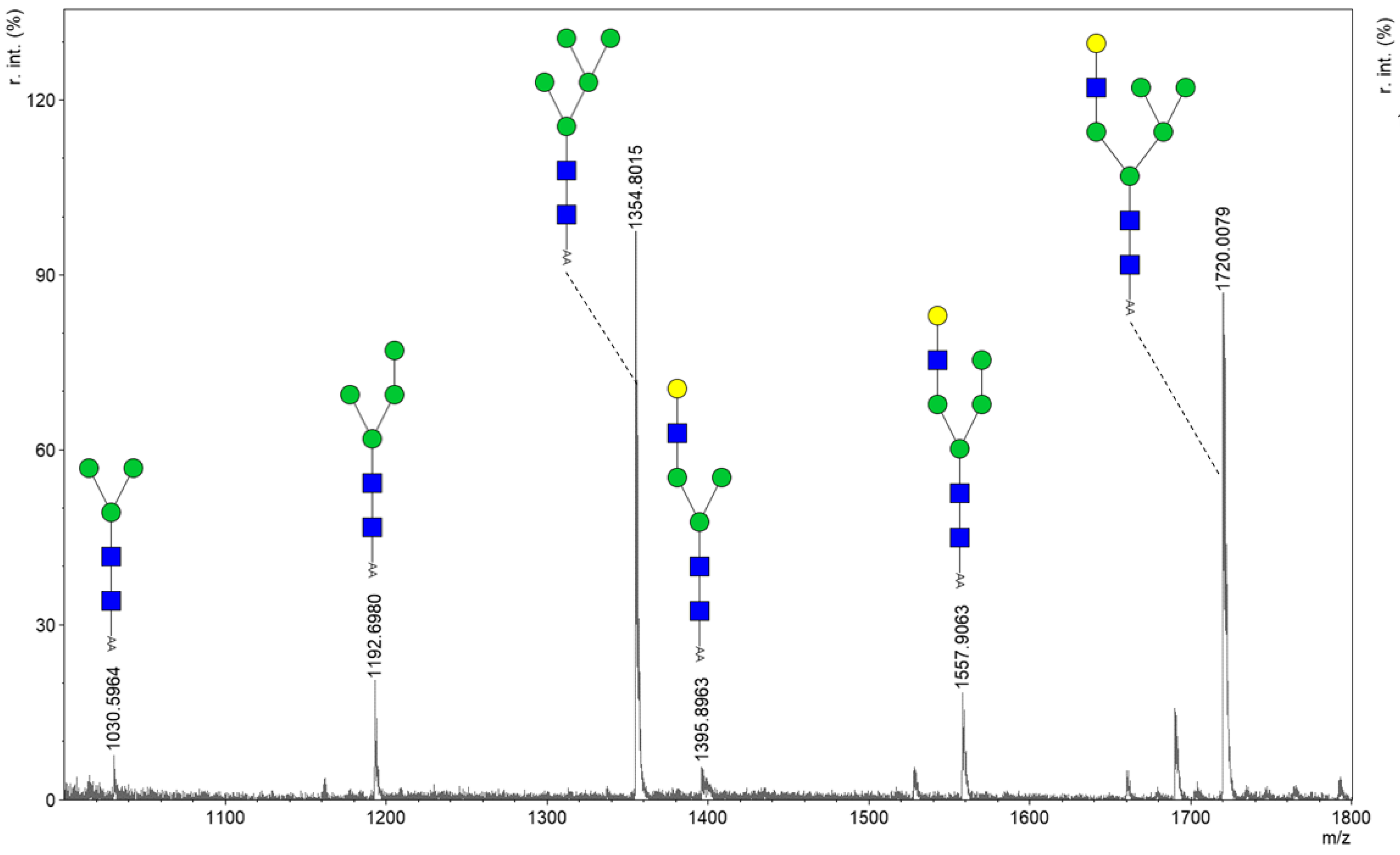

H

SmKappa-5 + 35S:SialDrGalT + NbBGAL17

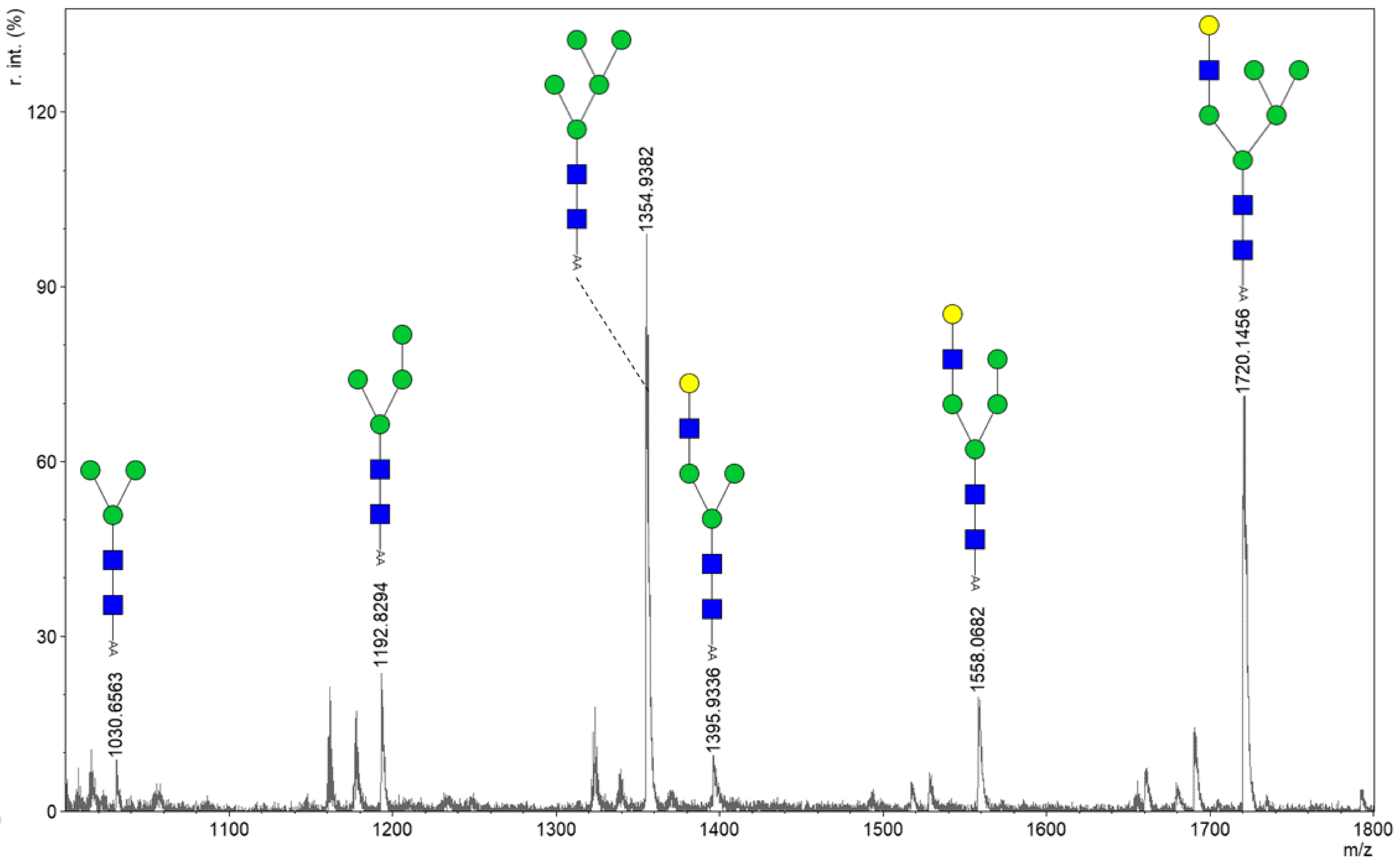

Supplemental figure 2. Cleavage of terminal β1,4-linked galactose from N-glycan by NbBGALs. Continued

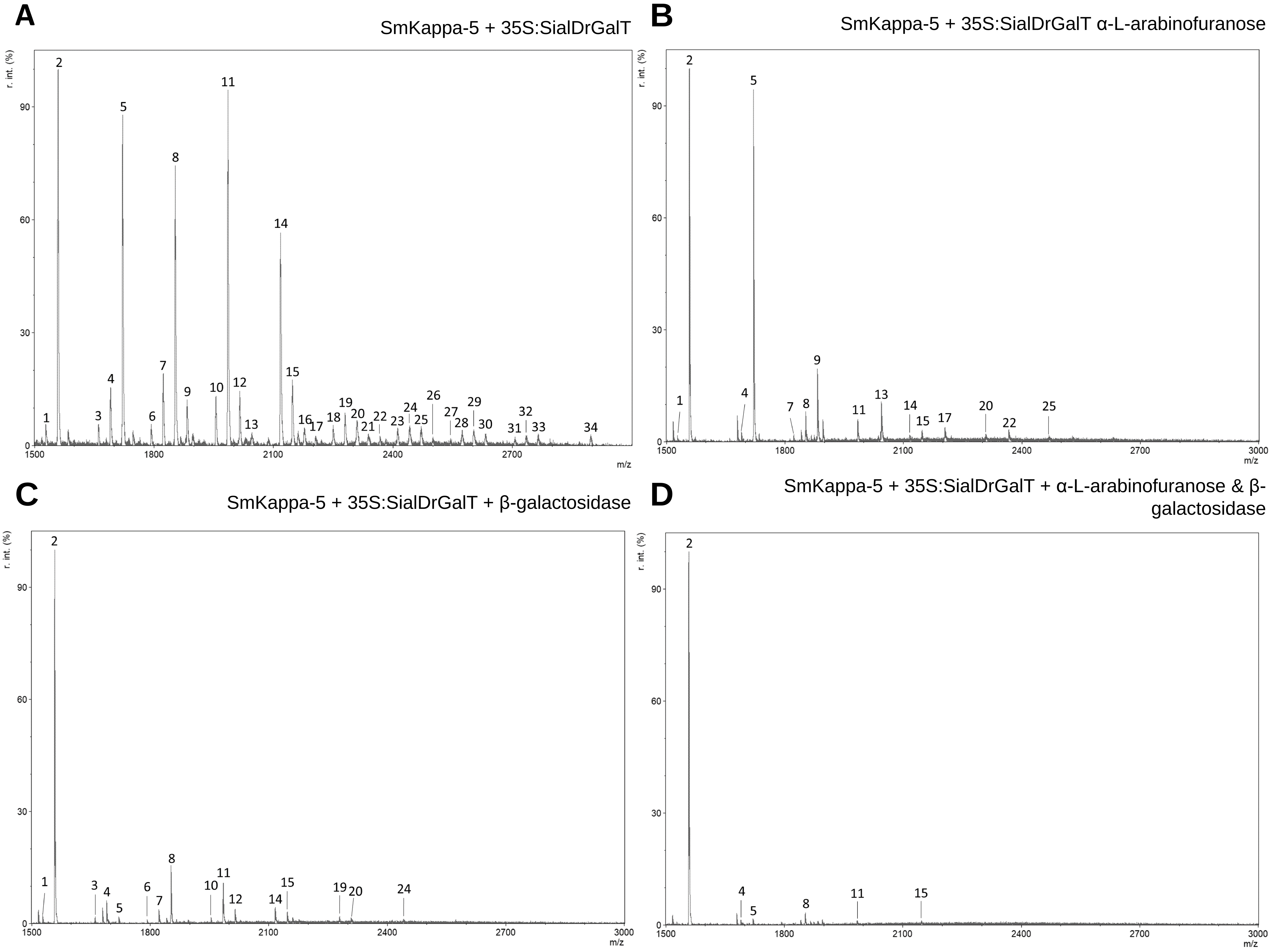

E

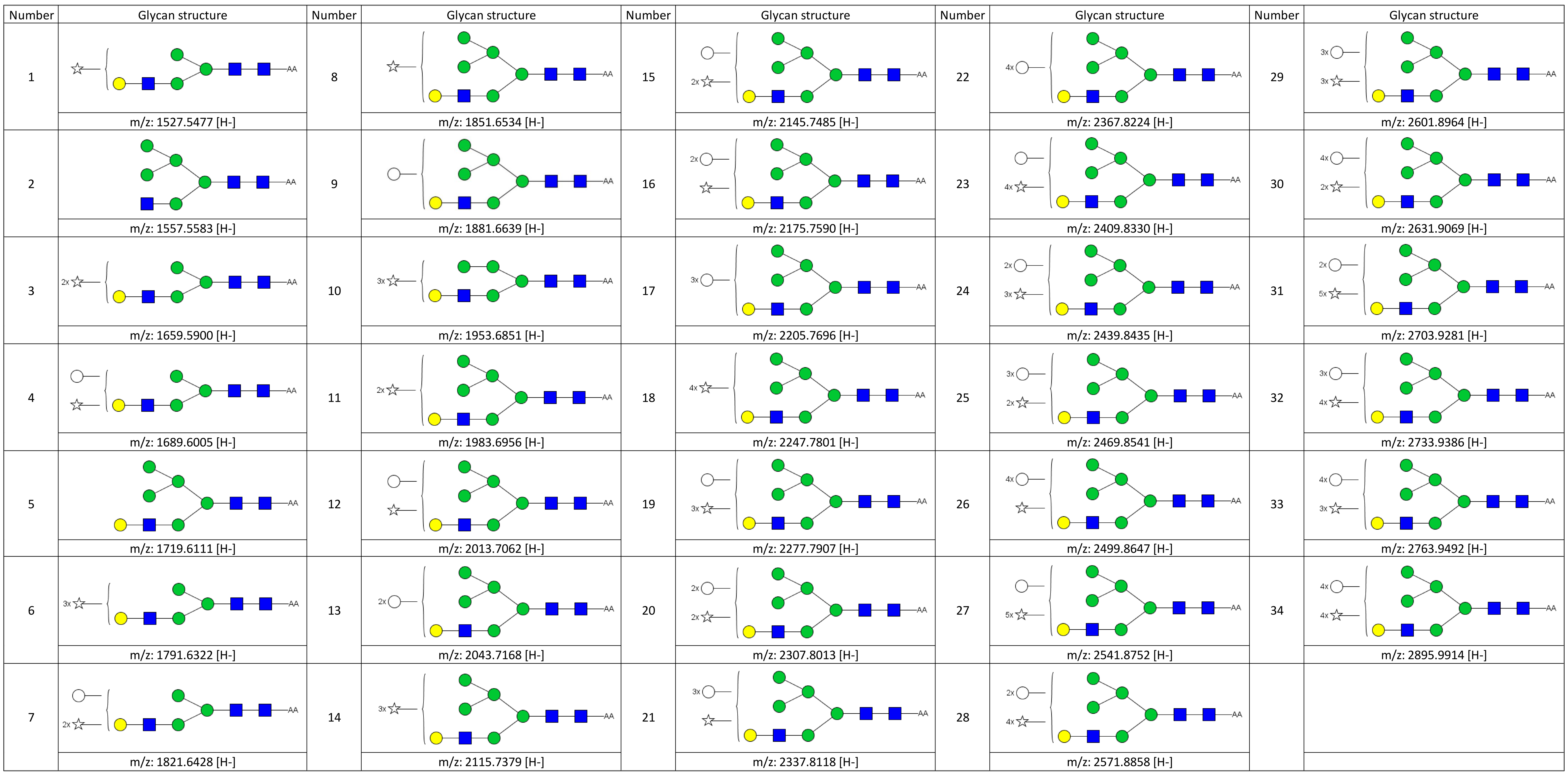

Number	Glycan structure	Number	Glycan structure	Number	Glycan structure	Number	Glycan structure	Number	Glycan structure
1	m/z: 1527.5477 [H-]	8	m/z: 1851.6534 [H-]	15	m/z: 2145.7485 [H-]	22	m/z: 2367.8224 [H-]	29	m/z: 2601.8964 [H-]
2	m/z: 1557.5583 [H-]	9	m/z: 1881.6639 [H-]	16	m/z: 2175.7590 [H-]	23	m/z: 2409.8330 [H-]	30	m/z: 2631.9069 [H-]
3	m/z: 1659.5900 [H-]	10	m/z: 1953.6851 [H-]	17	m/z: 2205.7696 [H-]	24	m/z: 2439.8435 [H-]	31	m/z: 2703.9281 [H-]
4	m/z: 1689.6005 [H-]	11	m/z: 1983.6956 [H-]	18	m/z: 2247.7801 [H-]	25	m/z: 2469.8541 [H-]	32	m/z: 2733.9386 [H-]
5	m/z: 1719.6111 [H-]	12	m/z: 2013.7062 [H-]	19	m/z: 2277.7907 [H-]	26	m/z: 2499.8647 [H-]	33	m/z: 2763.9492 [H-]
6	m/z: 1791.6322 [H-]	13	m/z: 2043.7168 [H-]	20	m/z: 2307.8013 [H-]	27	m/z: 2541.8752 [H-]	34	m/z: 2895.9914 [H-]
7	m/z: 1821.6428 [H-]	14	m/z: 2115.7379 [H-]	21	m/z: 2337.8118 [H-]	28	m/z: 2571.8858 [H-]		

Supplemental figure 3. Enzymatic digestion of β1,4-galactose engineered N-glycans containing unknown glycan residues.
To validate the presence and identity of the additional hexose and pentose residues the N-glycans were treated with *Aspergillus niger* α-L-arabinofuranose and/or β-galactosidase and analyzed by MALDI-TOF-MS. MS profiles are given for SmKappa-5 upon co-expression of 35S:SialDrGalT without enzymatic treatment of the N-glycans **(A)**, or after treatment with α-L-arabinofuranose **(B)**, β-galactosidase **(C)**, or both **(D)**. A table representing the different structures (1-34) is presented as well **(E)**.

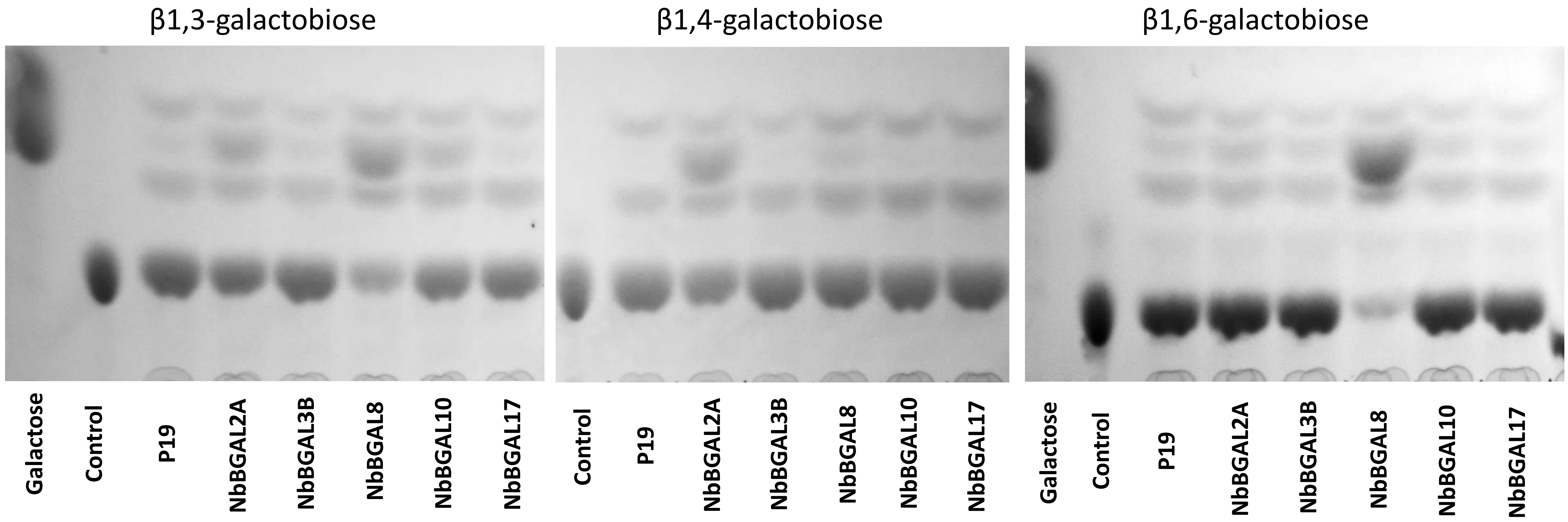

Supplemental figure 4: Enzymatic activity of NbGAL-mCitrine tagged enzymes against β1,3/β1,4/β1,6 linked galactobiose. Apoplast fluids from a P19 control infiltration or a selection of over-expressed NbBGAL-mCitrine fusions were incubated with galactobiose at pH 4.8 in sodium acetate buffer. Enzymatic activity was visualized with thin-layer chromatography versus a galactose and associated undigested galactobiose control.

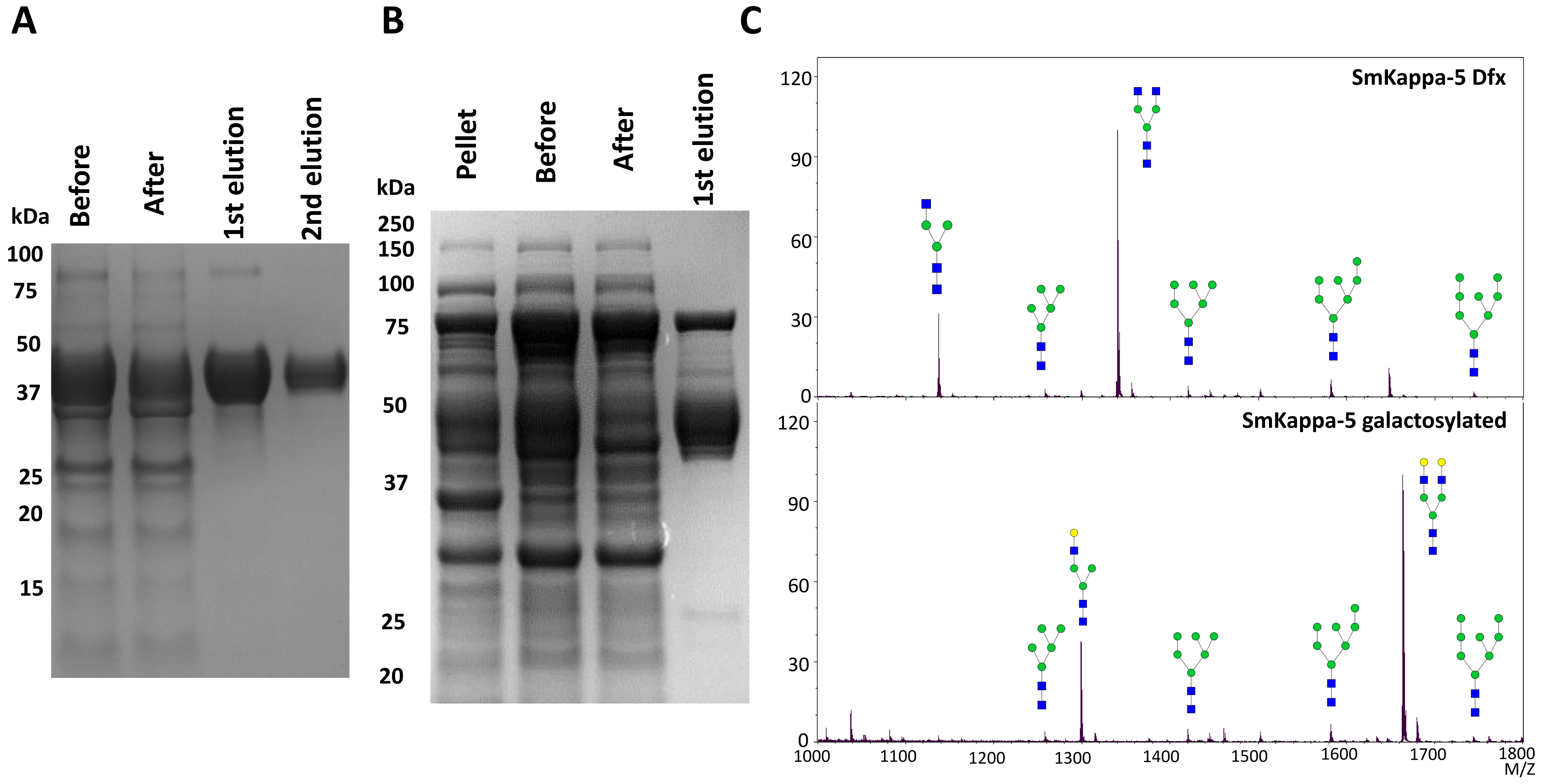

Supplemental figure 5. *In vitro* galactosylation of SmKappa-5 by MmGALT-6xHIS-MBP.
(A) SmKappa-5 was transiently overexpressed in ΔXT/FT *Nicotiana benthamiana* plants and purified from the apoplastic fluid via Ni-NTA Purification. **(B)** Codon optimized *Mus musculus* beta-1,4-galactosyltransferase 1 was expressed in the *E. coli* BL21 line, tagged with a N-terminal 6xhistidine and maltose binding protein (MBP) for enhanced solubility. MmGALT-6xHIS-MBP was purified from *E. coli* via Ni-NTA Purification. **(C)** Purified SmKappa-5 was galactosylated *in vitro* with MmGALT-6xHIS-MBP. N-glycans were released with PNGase F and analyzed with MALDI-TOF. The presented profiles show that SmKappa-5 is fully galactosylated after the enzymatic reaction.

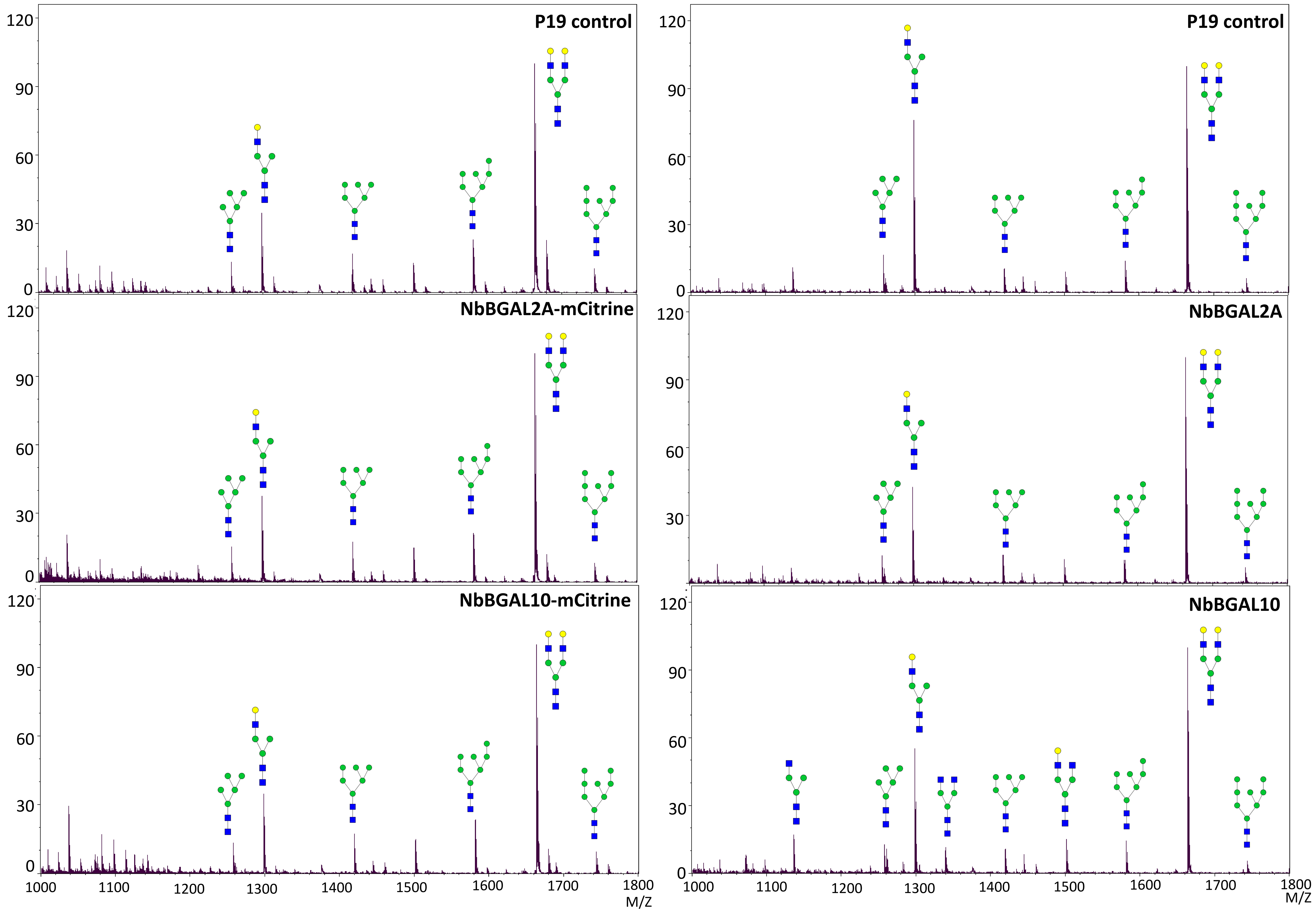

Supplemental figure 6: *In vitro* β-galactosidase activity of NbBGALs.
MALDI-TOF-MS N-glycan analysis of *in vitro* glyco-engineered SmKappa-5 after treatment with apoplast fluids from p19 control infiltrations or apoplast fluids upon overexpression of NbBGAL2A or NbBGAL10 (with or without mCitrine fusion).

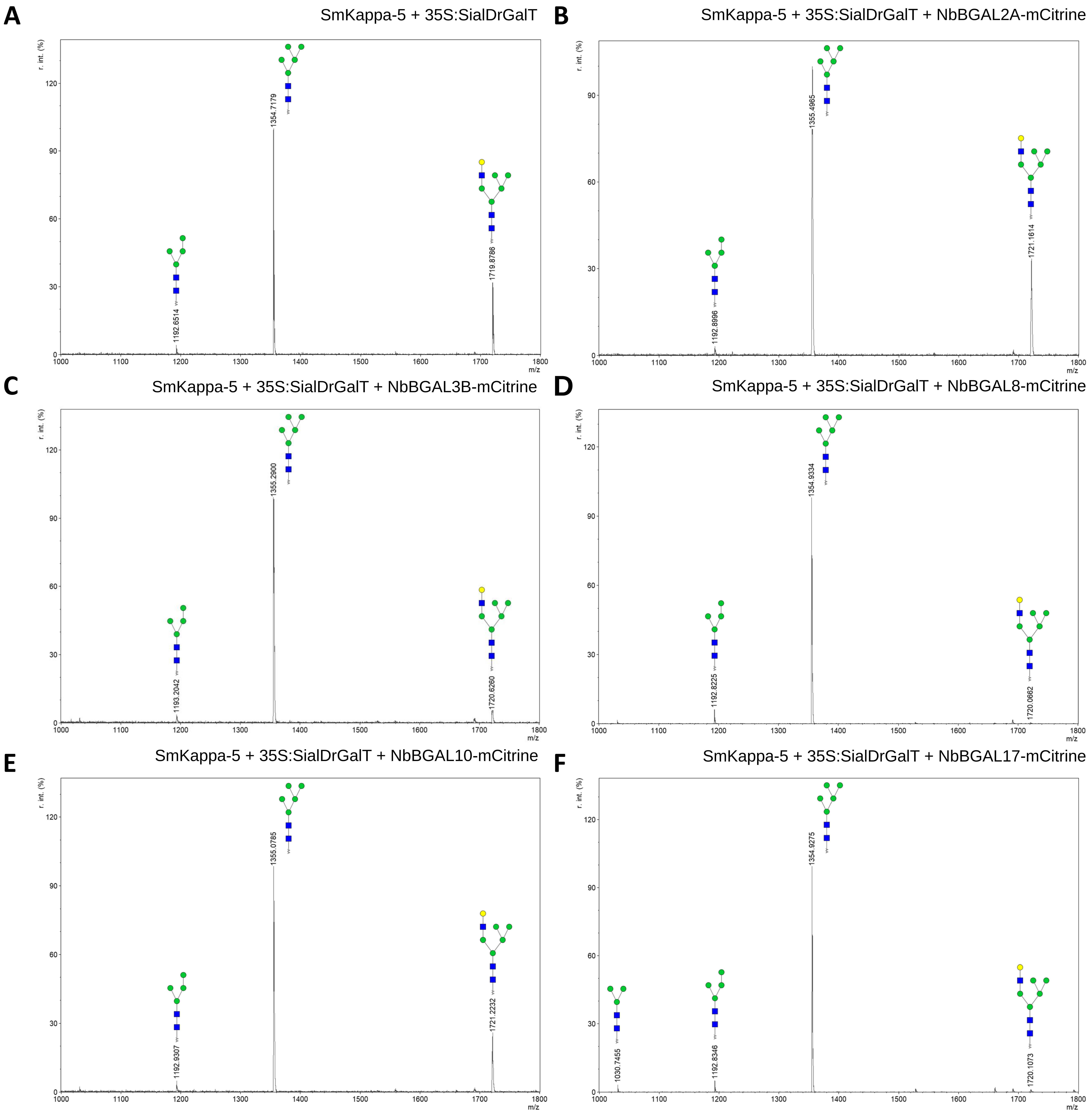

Supplemental figure 7. Cleavage of terminal β1,4-linked galactose from N-glycan by NbBGAL-mCitrine fusions.
SmKappa-5 was co-expressed with 35S:SialDrGalT and different NbBGALs fusion proteins in ΔXT/FT *N. benthmiana* plants. MALDI-TOF-MS N-glycan analysis of purified SmKappa-5 upon co-expression of 35S:SialDrGalT only **(A)**, or in combination with NbBGAL2A-mCtrine **(B)**, NbBGAL3B-mCitrine **(C)**, NbBGAL8 –mCitrine **(D)**, NbBGAL10-mCitrine **(E)**, or NbBGAL17-mCitrine **(F)**. All samples were treated with β-N-acetylglucosaminidase to confirm the presence of galactose-extended antenna. Peaks of interest were labeled with the corresponding N-glycan structures.

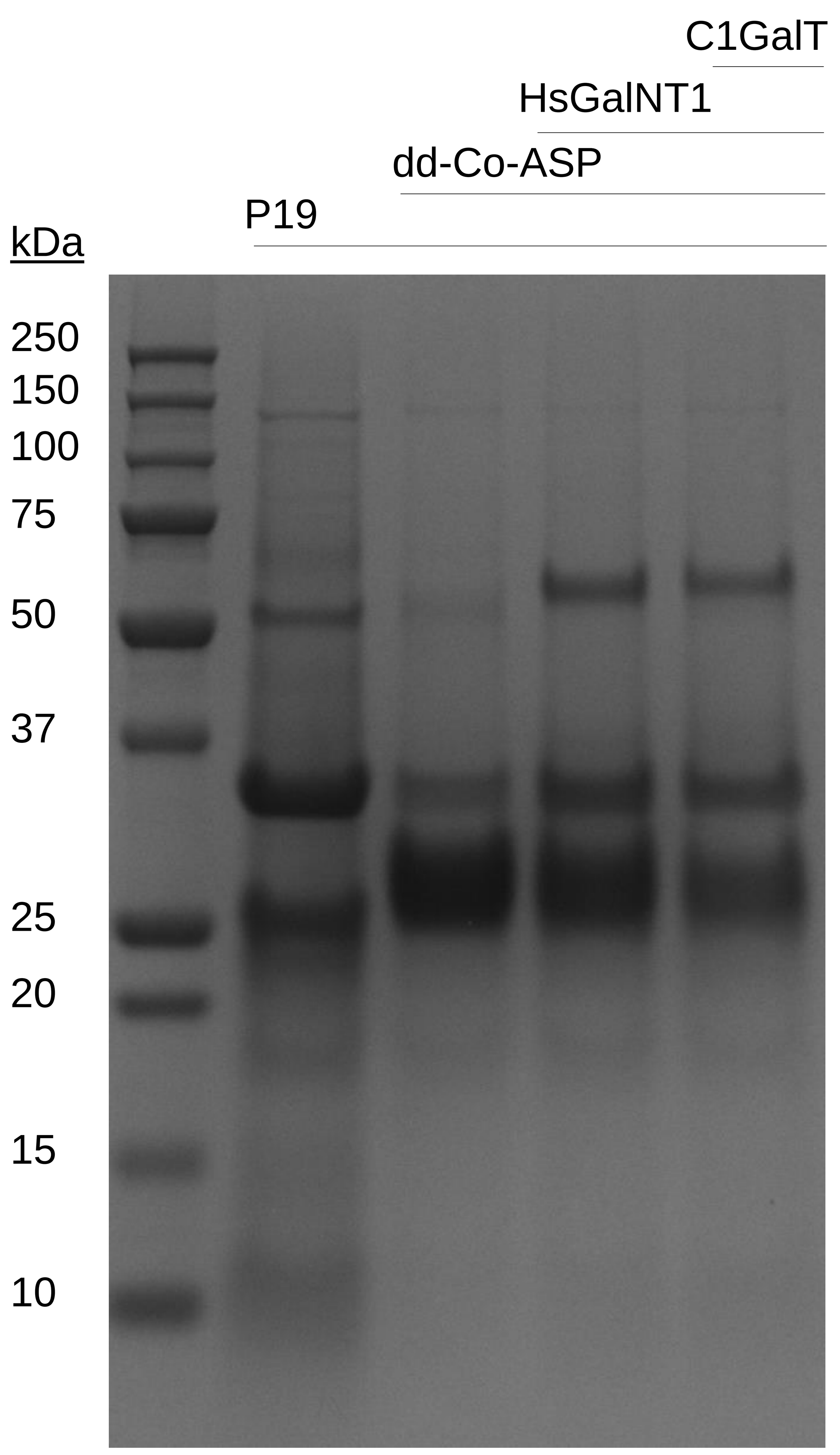

Supplemental figure 8. Expression of dd-Co-ASP in *N. benthamiana.*
SDS-PAGE and Coomassie blue staining of apoplast fluids of dd-Co-ASP co-expressed with HsGalNT1 and C1GalT. P19 was included in all samples to enhance expression. Intact dd-Co-ASP can be observed at ±51 kDa and cleavage products dd-Co-ASP can be observed at ±27 kDa. Introduction of O-glycans on dd-Co-ASP withHsGalNT1 seems to partially prevent cleavage.

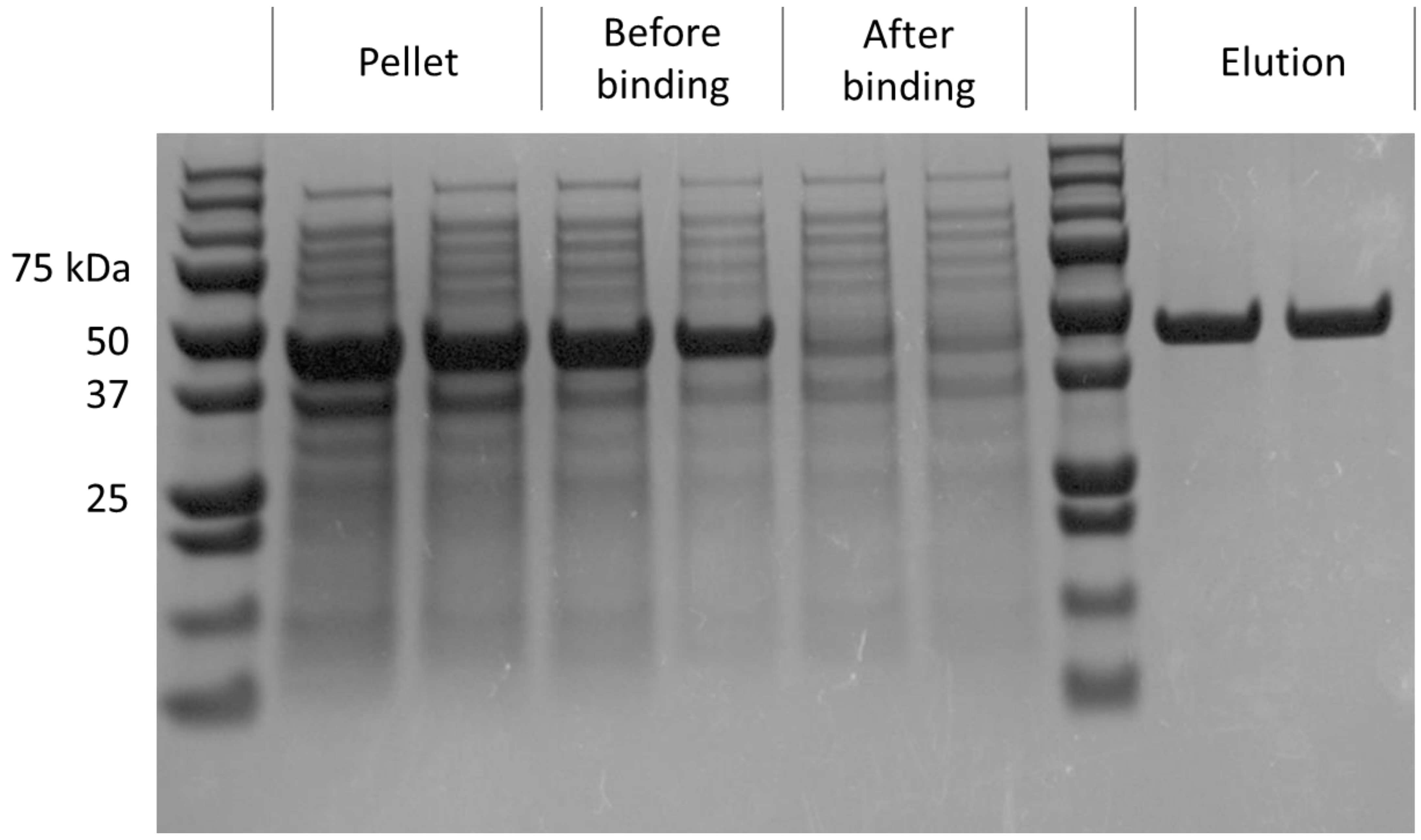

Supplemental Figure 9. SDS-PAGE analysis of production and purification of fucosidase isoform 3066 of bacterium *E. oligotrophica*.

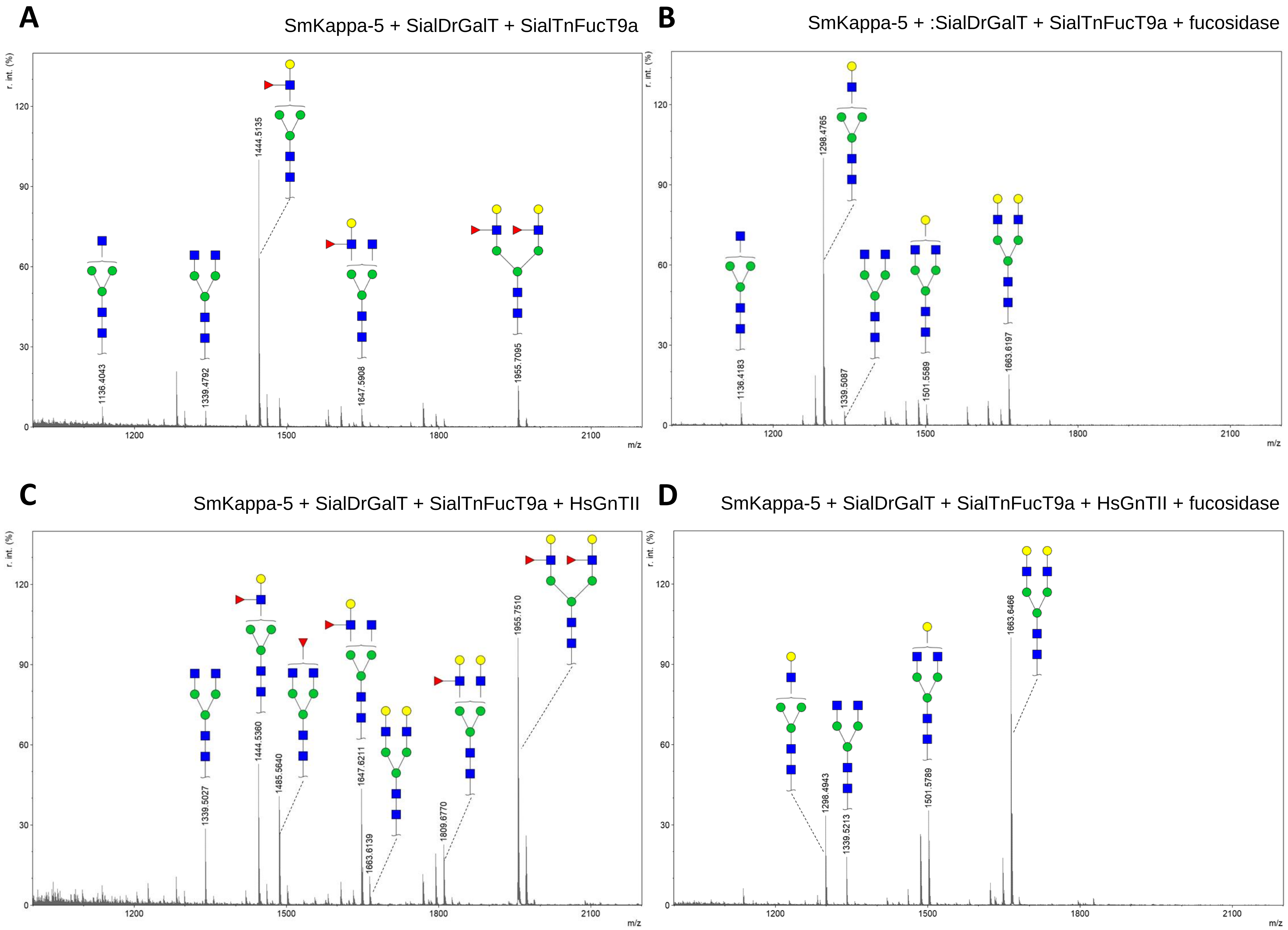

Supplemental Figure 10. Efficient galactosylation through Lewis X engineering in *N. benthamiana* and *in vitro* removal of antennary fucose.

SmKappa-5 was co-expressed with GPAII:sialDrGalT and SialTnFucT9a in ΔXT/FT *N. benthamiana* plants to enable Lewis X synthesis on N-glycans of SmKappa-5. **(A-B)** MALDI-TOF MS N-glycan profiles of purified Oo-ASP-1 upon co-expression of GPAII:SialDrGalT and SialTnFucT9a before (A) and after (B) *in vitro* fucosidase treatment. **(C-D)** MALDI-TOF MS N-glycan profiles of SmKappa-5 upon co-expression of GPAII:SialDrGalT, SialTnFucT9a and HsGnTII (for synthess of diantennary N-glycans) before (C) and after (D) *in vitro* fucosidase treatment.

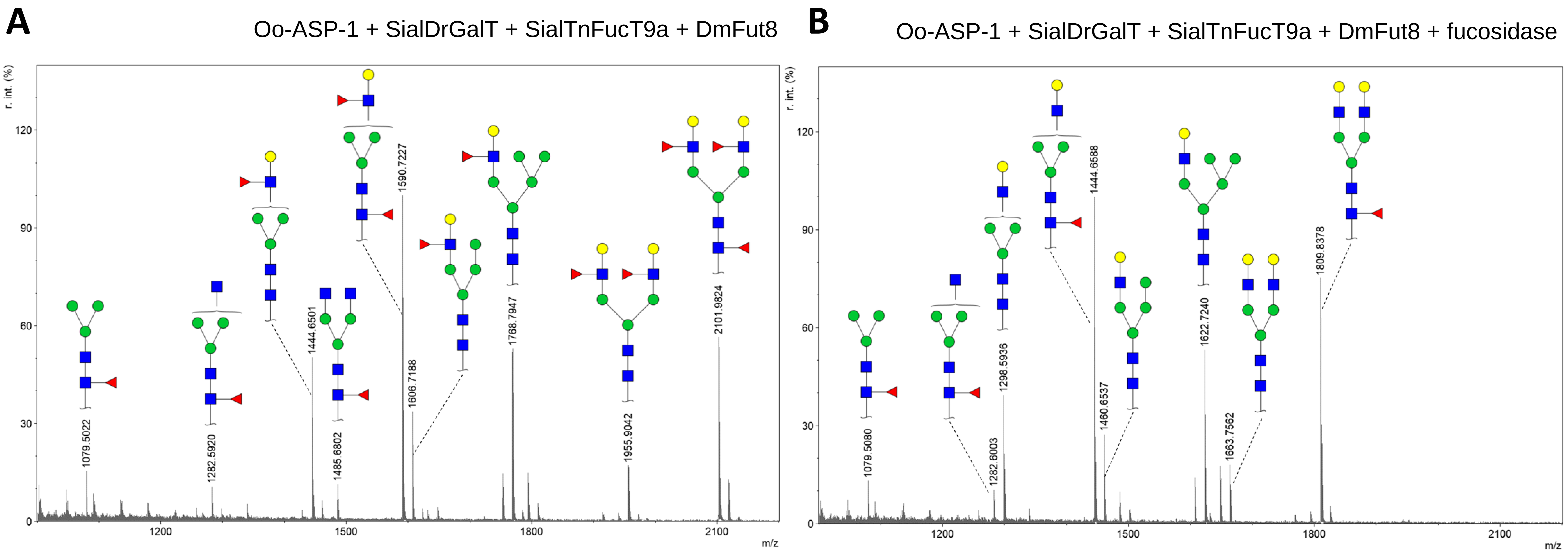

Supplemental Figure 11. *In vitro* fucosidase treatment of Lewis X engineered N-glycans does not remove the core α1,6-fucose on Oo-ASP-1 N-glycans.

Oo-ASP-1 was co-expressed with GPAII:sialDrGalT and SialTnFucT9a in ΔXT/FT *N. benthamiana* plants to enable Lewis X synthesis as well as DmFUT8 for core a1,6-fucosylation. **(A-B)** MALDI-TOF MS N-glycan profiles of Oo-ASP-1 upon co-expression sialDrGalT, TnFucT9a and DmFUT8 before (A) and after (B) *in vitro* fucosidase treatment.

Supplemental table 1: Primers used for cloning the NbBGAL overexpression constructs

Gene		Sequence (5'->3')	Restriction site	Accession number (Genbank)
NbBGAL 1	Fw	TCTAGAATGCTGACCAACATGGATTGTTCTTGGATTGC	XbaI	PQ189759
NbBGAL 1	Rv	GGATCCTCAACTGCAAATGGCTTCCACTGAGAGTTTC	BamHI	
NbBGAL 2a	Fw	GGATCCGCACCTTTCGCCTTCTTCTGTTTCTTTCTCCT	BamHI	PQ189760
NbBGAL 2a	Rv	GGGCCCCCCGTCCAAAGGAATTCAGGCACC	ApaI	
NbBGAL 3a	Fw	ccggatccATGGAAGTTAACACAGTTAAGAAGTGGG	BamHI	PQ189761
NbBGAL 3a	Rv	ggtgtacaTCAATTCTGGGTAGGaGTACAATGTGCTTC	BsrGI	
NbBGAL 3b	Fw	GGATCCATGGGTGCCAAATGGTTATTGCTATTCTTTGTTTTG	BamHI	PQ189762
NbBGAL 3b	Rv	GGGCCCCTATACATATGTAATTGATCTTTTGACAAGAGAAATCTTTGATGCATC	ApaI	
NbBGAL 8	Fw	CCTCATGAGATCTATGGGTTTATCCGTAATGCTAGTG	PagI	PQ189763
NbBGAL 8	Rv	GGGGTACCTCACGAACAGGAAGCTTCTACG	KpnI	
NbBGAL 8-L	Fw	GGATCCATGGAAAGAAAGTTGTTACTATTAG	BamHI	PQ189764
NbBGAL 8-L	Rv	TCTAGATCAAACACTGCAAAAAGCTTC	XbaI	
NbBGAL 9a	Fw	ccggatccATGGTGATCAGAAGAAAAGCAGCTC	BamHI	PQ189765
NbBGAL 9a	Rv	ggggtaccTCACACGGAAGCTGAAGCG	KpnI	
NbBGAL 10	Fw	TCTAGAGGCAATGGTGCTCAGCTGC	XbaI	PQ189766
NbBGAL 10	Rv	GGATCCCGTGGGGGTTGAAATACAATGAGTAC	BamHI	
NbBGAL 16a	Fw	GGATCCGTGCGTATGTGTGTTTGGATGGTGG	BamHI	PQ189777
NbBGAL 16a	Rv	GGATCCGCATTTCGCCTGCCCTAAACAGG	XbaI	
NbBGAL 17	Fw	GGATCCCTTCAACTTTCAATGACCACAACGG	BamHI	PQ189778
NbBGAL 17	Rv	TCTAGACAAAGACATGATGATGTCAGATTTGATGC	XbaI	

Supplemental table 2: Primers used for NbBGAL-mCitrine fusion cloning

NbBGAL1B mCit FW	**tcaaatacTTCCACCGGATCC**ATGCTGACCAACATGGATT
NbBGAL1B mCit RV	**ggaaccaccaccaccACTGCAAATGGCTTCCA**
NbBGAL2A mCit FW	**tcaaatacTTCCACCGGATCC**ATGCTAAGCACCAATGTATTATTG
NbBGAL2A mCit RV	**ggaaccaccaccaccTCTTGATCTTCCGACCAAAG**
NbBGAL3A mCit FW	**tcaaatacTTCCACCGGATCC**ATGGAAGTTAACACAGTTAAGAAGT
NbBGAL3A mCit RV	**ggaaccaccaccacc**ATTCTGGGTAGGAGTACAATGT
NbBGAL3B mCit FW	**tcaaatacTTCCACC**ATGGGTGCCAAATGGTTA
NbBGAL3B mCit RV	**ggaaccaccaccacc**TACATATGTAATTGATCTTTTGACAAG
NbBGAL8 mCit FW	**tcaaatacTTCCACC**ATGGGTTTATCCGTAATG
NbBGAL8 mCit RV	**ggaaccaccaccacc**CGAACAGGAAGCTTCTAC
NbBGAL8-L mCit FW	**tcaaatacTTCCACCGGATCC**ATGGAAAGAAAGAAGTTGTTACTATTAG
NbBGAL8-L mCit RV	**ggaaccaccaccacc**AACACTGCAAAAAGCTTCAA
NbBGAL9A mCit FW	**tcaaatacTTCCACCGGATCC**ATGGTGATCAGAAGAAAAGC
NbBGAL9A mCit RV	**ggaaccaccaccacc**CACGGAAGCTGAAGTGCT
NbBGAL10 mCit FW	**tcaaatacTTCCACCGGATCC**ATGAAGACGATGAGTCATTTCT
NbBGAL10 mCit RV	**ggaaccaccaccacc**GCTGCAATTTACTTCAACGG
NbBGAL16A mCit FW	**tcaaatacTTCCACCGGATCC**ATGAGAATACAGTGTCTATGGC
NbBGAL16A mCit RV	**ggaaccaccaccacc**TATAAAACTTGTGTTATTATTAAGGGTAGATTT
NbBGAL17 mCit FW	**tcaaatacTTCCACCGGATCC**ATGACCACAACGGTGAAG
NbBGAL17 mCit RV	**ggaaccaccaccacc**GATTTGATGCACTTTTGAATTTGC

**Supplemental table 3: Primers used for enzyme production in *E. coli***	
pCOLD B4GALT FW	taggtaatctctgcttaaaagcacag
pCOLD B4GALT RV	cactttgtgattcatggtgtatt
B4GALT FW	CACCATCACCATCATCACGGAAGAGATTTATCAAGGCT
B4GALT RV	ttaagcagagattacctaTCTCGGAGTGCCGATGTC
MBP FW	TGATGATGGTGATGGTGGCTAGCCATcgaattagtctgcgcgtc
MBP RV	atgaatcacaaagtggaagaaggtaaactggtaatctggat
EO3066 FW	CACCATCACCATCATCACGCAACGCCATCACCTGAC
EO3066 RV	ttaagcagagattacctaCTCAGGCGTATTATACAATTC
pCOLD EO3066 FW	taggtaatctctgcttaa
pCOLD EO3066 RV	GTGATGATGGTGATGGTGcactttgtgattcatggt

Supplemental table 4: Characterized activity of recombinantly produced radish, tomato and Arabidopsis β-galactosidases

Gene ID	NbBGAL ortholog	β1,3 galactosidase	β1,4 galactosidase	β1,6 galactosidase	Reference
**RsBGAL1**	NbBGAL8	X		X	Kotake et al, 2005
**TBG4**	NbBGAL2A	X	X		Ishimaru et al, 2009
**TBG5**	NbBGAL8	X		X	Ishimaru et al, 2009
**AtBGAL2**	NbBGAL2A	X	X		Gantulga et al, 2008
**AtBGAL3**	NbBGAL3B	X	X		Gantulga et al, 2009
**AtBGAL4**	NbBGAL4	X	X		Gantulga et al, 2009
**AtBGAL5**	NbBGAL5	X	X		Gantulga et al, 2008
**AtBGAL12**	NbBGAL12	X	X	X	Gantulga et al, 2009
